# Divergent evolutionary and epidemiological dynamics of cassava mosaic geminiviruses in Madagascar

**DOI:** 10.1186/s12862-016-0749-2

**Published:** 2016-09-06

**Authors:** Alexandre De Bruyn, Mireille Harimalala, Innocent Zinga, Batsirai M. Mabvakure, Murielle Hoareau, Virginie Ravigné, Matthew Walters, Bernard Reynaud, Arvind Varsani, Gordon W. Harkins, Darren P. Martin, Jean-Michel Lett, Pierre Lefeuvre

**Affiliations:** 1 CIRAD, UMR PVBMT, Pôle de Protection des Plantes, 7 chemin de l’IRAT, Saint-Pierre, Ile de la Réunion 97410 France; 2 Université de la Réunion, UMR PVBMT, Pôle de Protection des Plantes, 7 chemin de l’IRAT, Saint-Pierre, Ile de la Réunion 97410 France; 3 FOFIFA, Laboratoire de Pathologie Végétale, BP 1444 Ambatobe, Madagascar; 4 LSBAD, Université de Bangui, BP908 Bangui, Centrafrique France; 5 South African National Bioinformatics Institute, Medical Research Council Bioinformatics Unit, University of the Western Cape, Cape Town, South Africa; 6 CIRAD, UMR BGPI, Campus International de Baillarguet, Montpellier, 34398 France; 7 School of Biological Sciences and Biomolecular Interaction Centre, University of Canterbury, Private Bag 4800, Christchurch, New Zealand; 8 Department of Plant Pathology and Emerging Pathogens Institute, University of Florida, Gainesville, FL 32611 USA; 9 Structural Biology Research Unit, University of Cape Town, Rondebosch, 7701 Cape Town, South Africa; 10 Institute of Infectious Disease and Molecular Medicine, University of Cape Town, Observatory 7925, Cape Town, South Africa

**Keywords:** Begomoviruses, Cassava, Epidemiology, Madagascar, Phylogeography, Recombination

## Abstract

**Background:**

Cassava mosaic disease (CMD) in Madagascar is caused by a complex of at least six African cassava mosaic geminivirus (CMG) species. This provides a rare opportunity for a comparative study of the evolutionary and epidemiological dynamics of distinct pathogenic crop-infecting viral species that coexist within the same environment. The genetic and spatial structure of CMG populations in Madagascar was studied and Bayesian phylogeographic modelling was applied to infer the origins of Madagascan CMG populations within the epidemiological context of related populations situated on mainland Africa and other south western Indian Ocean (SWIO) islands.

**Results:**

The isolation and analysis of 279 DNA-A and 117 DNA-B sequences revealed the presence in Madagascar of four prevalent CMG species (*South African cassava mosaic virus*, SACMV; *African cassava mosaic virus*, ACMV; *East African cassava mosaic Kenya virus*, EACMKV; and *East African cassava mosaic Cameroon virus*, EACMCV), and of numerous CMG recombinants that have, to date, only ever been detected on this island. SACMV and ACMV, the two most prevalent viruses, displayed low degrees of genetic diversity and have most likely been introduced to the island only once. By contrast, EACMV-like CMG populations (consisting of *East African cassava mosaic virus*, EAMCKV, EACMCV and complex recombinants of these) were more diverse, more spatially structured, and displayed evidence of at least three independent introductions from mainland Africa. Although there were no statistically supported virus movement events between Madagascar and the other SWIO islands, at least one mainland African ACMV variant likely originated in Madagascar.

**Conclusions:**

Our study highlights both the complexity of CMD in Madagascar, and the distinct evolutionary and spatial dynamics of the different viral species that collectively are associated with this disease. Given that more distinct CMG species and recombinants have been found in Madagascar than any other similarly sized region of the world, the risks of recombinant CMG variants emerging on this island are likely to be higher than elsewhere. Evidence of an epidemiological link between Madagascan and mainland African CMGs suggests that the consequences of such emergence events could reach far beyond the shores of this island.

**Electronic supplementary material:**

The online version of this article (doi:10.1186/s12862-016-0749-2) contains supplementary material, which is available to authorized users.

## Background

Amongst the many known plant pathogens, viruses are responsible for the majority of the emerging diseases that threaten food production worldwide [1]. However, viruses in their native environments rarely cause damaging diseases [2]. Within the undisturbed ecological contexts of such environments, the numerous interactions that viruses encounter with their natural host and transmission vector species are generally both evolutionarily ancient and relatively stable [3]. The rise of modern agriculture has been accompanied by the dissemination of large numbers of exotic plant species, transmission vectors and viruses into foreign environments, which has precipitated multitudes of novel evolutionarily recent virus-host-vector-environment interactions. It is possible that the instability of some of these “unnatural” interactions, has in many cases triggered the emergence of devastating new viral diseases [2].

In this regard, the cultivation of cassava in Africa represents an excellent example both of how the introduction of exotic plant species into foreign ecosystems can provide opportunities for novel interactions, and of how such interactions can have far-reaching socio-economic consequences. Following two independent introductions of cassava from the Americas onto the African continent in the 16th and 18th centuries [4], cultivation of this crop subsequently spread throughout tropical Africa to the point where it has today become a major source of dietary carbohydrates for over 500 million Africans [5]. Concomitant with the intensification of cassava cultivation in Africa has been the emergence of cassava mosaic disease (CMD). Caused by a diverse group of at least nine distinct native African virus species that are collectively referred to as cassava mosaic geminiviruses (CMGs, genus: *Begomovirus*; family: *Geminiviridae*), CMD has evolved to become one of the most socio-economically damaging crop pathogens to have ever existed.

All known CMG species (Additional file 1: Figure S1), including *African cassava mosaic virus* (ACMV, [6, 7]), *East African cassava mosaic virus* (EACMV, [8]), *East African cassava mosaic Cameroon virus* (EACMCV, [9]), *East African cassava mosaic Malawi virus* (EACMMV, [10]), *East African cassava mosaic Zanzibar Virus* (EACMZV, [11]), *South African cassava mosaic virus* (SACMV, [12]), *East African cassava mosaic Kenya virus* (EACMKV, [13]), African cassava mosaic Burkina Faso virus (ACMBFV, [14]) and Cassava mosaic Madagascar virus (CMMGV, [15]), have genomes consisting of two components, called DNA-A and DNA-B, each comprising a 2.7 kb circular single-stranded DNA molecule [6]. All of these viruses are transmitted by *Bemisia tabaci*: a whitefly species complex consisting of several cryptic species [16].

A particularly interesting feature of the known CMG species is that they apparently have broadly overlapping geographical ranges (Additional file 1: Figure S1), they frequently co-occur within mixed infections [17–22], they readily exchange genetic material through homologous recombination [23, 24] and genome reassortment [20, 25], and they appear to synergistically interact with one another to cause infections with increased severity [20]. For example, the extremely severe CMD epidemic that has devastated cassava production in East Africa since the early 1990s was initiated by the simultaneous emergence of an ultra-pathogenic, apparently synergistic, combination of ACMV and a recombinant EACMV variant (called the EACMV-Uganda strain, [26]), and an increase in the density of *B. tabaci* populations [27].

A variety of different factors may have facilitated the initial emergence of CMGs from what were presumably viruses adapted to native African plant species. Among these are: (1) the introduction into Africa of a *B. tabaci* cryptic species with a larger host range than native African *B. tabaci* species and which may have in turn contributed to increased rates of begomovirus transmission from uncultivated hosts into cassava, and from cassava to cassava [27, 28]; (2) the high mutation [29] and recombination rates [23, 24] of begomoviruses which may have enabled the rapid adaptation of native African viruses to infecting cassava; and (3) the widespread practice of vegetatively propagating cassava which may have vastly increased the rates of human-mediated CMG dispersal in tropical Africa [30–32].

A comparative study of the respective evolutionary and migration histories of distinct and potentially interacting viral species sharing the same host and vector, such as the CMGs, could provide valuable insights into the host, vector or viral genetic factors that underlie the spatial and genetic structuring of virus populations, and the epidemiological factors that are associated with virus emergence. Indeed, a better understanding of such factors is key to identifying both the genetic features of virus populations that predispose some viral lineages to emerge as serious new crop pathogens, and the regions of the world where virus emergence events are most likely to occur.

Several studies of CMG diversity and evolution have been conducted in East Africa [13, 19, 33], West Africa [18, 19] and on the South West Indian Ocean islands [32]. Whereas these studies have revealed the extensive dissemination of different CMG species across Africa and the SWIO islands, each study focused primarily on only a small subset of the known CMG species: a factor which has made it difficult to compare the spatial distributions and evolutionary characteristics of the different CMGs.

In Madagascar, CMD arose as a major constraint on cassava cultivation during the 1930s in the central highlands and then spread within two years into all other cassava-growing regions of the island [34]. Despite the importance of the disease, molecular and epidemiological data on the causal species were lacking until 2012 when a series of surveys carried out between 2009 and 2011 [15, 22] indicated the presence on the island of six of the nine known African CMG species: a higher degree of CMG diversity than has been detected in any other part of the world. Importantly, these studies also indicated both that the six Madagascan CMG species likely have at least partially overlapping geographical ranges, and that frequent co-infections containing two or more species also occur. While the presence of such a high degree of CMG diversity in Madagascar suggests that multiple virus introduction events have likely occurred over the past century, the occurrence of overlapping geographical ranges and mixed infections suggests that many of these viruses are directly interacting with one-another. In this regard, Madagascar is in many ways a microcosm of Africa that offers an ideal opportunity to comparatively study the movement and evolutionary dynamics of multiple viral species that infect the same hosts and coexist within an enclosed but relatively large geographical area.

In this study we characterized a total of 279 CMG DNA-A sequences and 117 CMG DNA-B sequences sampled from a range of locations throughout Madagascar. We investigated the genetic diversity and distributions of the six CMG species infecting cassava crops on the island, and attempted to reconstruct their evolutionary histories within a spatio-temporal context.

## Results

### The occurrence of six CMG species in Madagascar

**Fig. 1:**
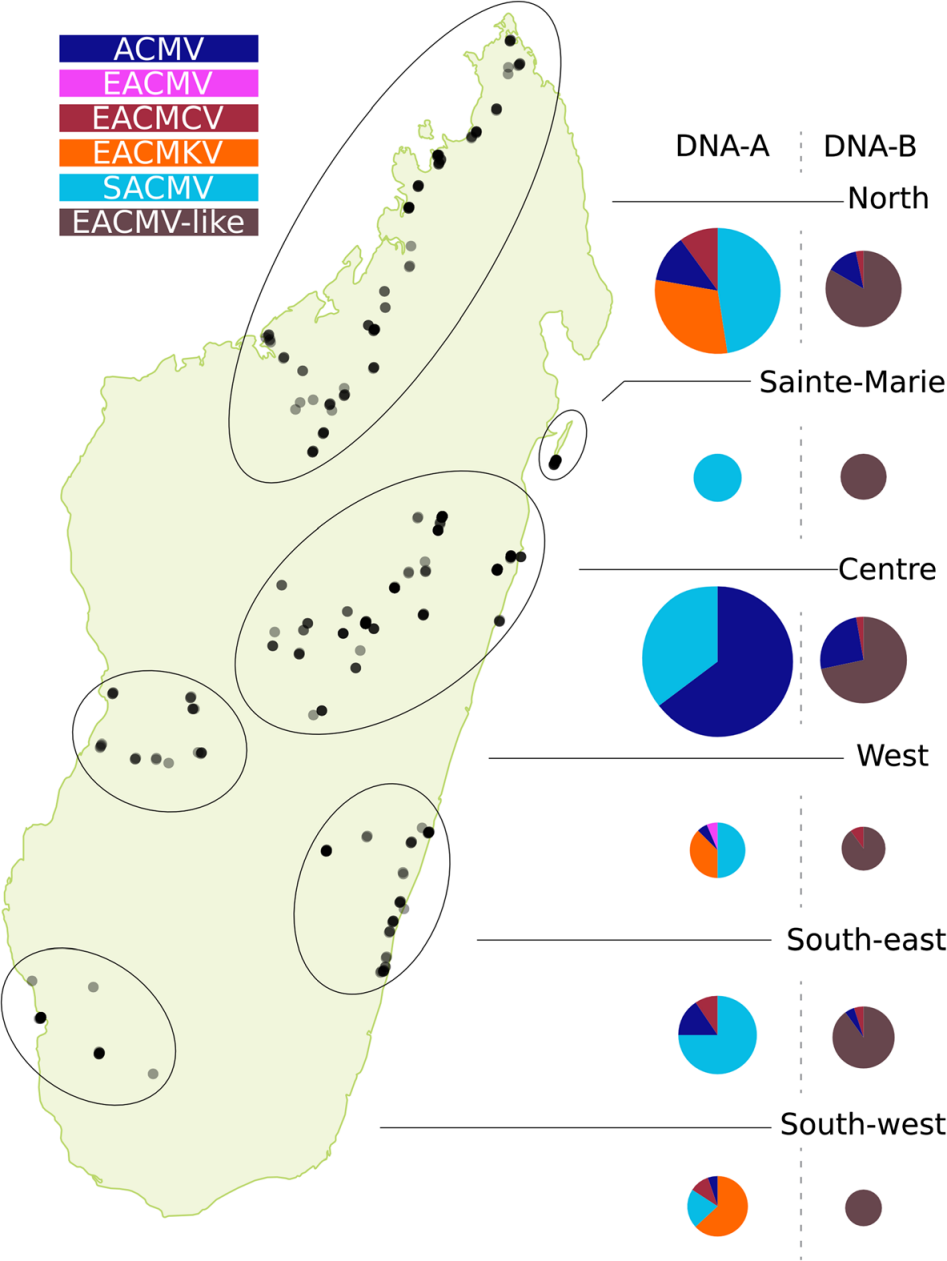
Species composition and repartition map of the CMGs in Madagascar. *Black dots* indicate the location of samples from which sequences (DNA-A or DNA-B) were isolated, with the degree of transparency representing density of sequence sampling. Based on hierarchical clustering of the sample coordinates, five distinct sampled areas were defined, and are indicated by *grey ellipses*. The species composition of DNA-A and DNA-B are indicated for each area, with the corresponding colour code being given at the bottom of the figure

Besides PCR typing data on a large number of infected Madagascan cassava samples [22], prior to this study very little sequence information was available for CMGs circulating on the island. The information available in public sequence databases included only three complete CMG genome component sequences: the DNA-A component of a SACMV [GenBank:AJ422132] [35], and the DNA-A and DNA-B components of a novel recombinant begomovirus, CMMGV [15]. As part of this study, a total of 279 full DNA-A and 117 full DNA-B components were cloned and sequenced from 173 symptomatic Madagascan cassava leaves sampled during an epidemiological survey conducted between 2009 and 2011 [22] as well as 91 additional samples collected on the island between 2005 and 2008. Based on the 91 % DNA-A identity criterion used as a *Begomovirus* species demarcation threshold [36], these sequences were classified into five of the nine previously described CMG species: SACMV, ACMV, EACMKV, ECMCV and EACMV (Fig. 1). Despite the recent isolation of a single CMMGV genome from the south-western part of the island [15], no additional isolates of this species were discovered here.

SACMV and ACMV were the most prevalent of the CMGs, respectively accounting for 46 and 33 % of the sampled DNA-A sequences. For each of these two species, sequences from Madagascar were both very similar to one another, with respective mean identities of 98.5 and 97.0 % (Table 1), and were considered, under the recommended 94 % Begomovirus strain demarcation criterion [36], to belong to the same strain of their respective species circulating on mainland Africa (Additional file 2: Figure S2).

**Table 1:** Comparison of diversity, evolution rates and introduction history in Madagascar of the different CMG species

	ACMV DNA-A	ACMV DNA-B	SACMV DNA-A	EACMV DNA-A	EACMKV DNA-A	EACMCV DNA-A	EACMV-like DNA-B	EACMCV DNA-B
[FS] Total sequences	212	95	132	201	114	29	215	10
[FS] Total diversity (mean Id%)	96.6 % [85.9 %–100 %]	93.2 % [90.0 %–100 %]	98.6 % [90.1 %–100 %]	94.3 % [83.5 %–100 %]	95.7 % [84.3 %–100 %]	94.7 % [89.7 %–99.9 %]	92.2 % [87.3 %–100 %]	90.3 % [84.8 %–97.3 %]
[FS] MG sequences	93	15	130	1	43	13	98	4
[FS] MG diversity (mean Id%)	98.5 % [97.1 %–100 %]	97.1 % [95.5 %–99.9 %]	98.7 % [93.3 %–100 %]	/	94.4 % [84.3 %–100 %]	96.3 % [93.1 %–99.9 %]	97.6 % [90.3 %–100 %]	91.1 % [84.8 %–94.8 %]
[FS] MG detected recombinants (%)	0 (0 %)	0 (0 %)	8 (6 %)	1 (100 %)	17 (40 %)	10 (77 %)	2 (2 %)	1 (25 %)
[core CP] Total sequences	218	/	132	244			/	/
[core CP] Total diversity (mean Id%)	97.0 % [91.5 %–100 %]	/	98.5 % [80.3 %–100 %]	95.6 % [89.8 %–100 %]			/	/
[core CP] MG sequences	93	/	130	51			/	/
[core CP] MG diversity (mean Id%)	98.7 % [95.6 %–100 %]	/	98.8 % [82.1 %–100 %]	94.9 % [90.0 %–100 %]			/	/
Introduction events	1	1	/	3–4			1	/
Introduction dates (95 % HPD)	1996–2004 [1995–2005]	1940–1974 [1924–1986]	/	1988–1990 [1982–1997]			1961–1978 [1921–1989]	/
				1988–1996 [1983–2003]				
				1984–2003 [1971–2006]				
				1997–1999 [1994–2003]				
Substitution rates (subs/site/year)	3.83 × 10^−3^ [2.82 × 10^−3^; 4.89 × 10^−3^]	5.64 × 10^−4^ [4.13 × 10^−4^; 7.14 × 10^−4^]	/	1.69 × 10^−3^ [1.31 × 10^−3^ to 2.12 × 10^−3^]			1.10 × 10^−3^ [9.57 × 10^−4^; 1.28 × 10^−3^]	/

For each dataset, the total and Madagascan (MG) number of sequences, mean and range of identity percentages are indicated ([FS] = Full Sequence, [core CP] = core of the capsid protein encoding ORF), as well as the number of recombinant Madagascan sequences isolated in this study. The number and dates of inferred introduction events as well as the inferred substitution rates are based on analyses of the core CP datasets for DNA-A and on full component sequences for DNA-B. Correlation coefficients related to the temporal signal of each dataset are listed

In contrast to ACMV and SACMV isolates, the 43 Madagascan EACMKV isolates (accounting for 15 % of the sampled DNA-A sequences) and the 13 Madagascan EACMCV isolates (accounting for 4.6 % of the sampled DNA-A sequences) were more diverse, with mean DNA-A identities of 94.4 % (all >84.3 %) and 96.3 % (all >93.1 %), respectively (Additional file 2: Figure S2). Interestingly, one of the EACMKV isolates from the Diana region of Madagascar [GenBank:KJ888083] was <91 % identical to all other presently available isolates of this species but one (KJ888079 with 91.0 % identity). Whereas this isolate cannot be considered a member of a new species, it is the only one representing a novel strain that is possibly unique to the SWIO islands. With only one sequence represented in our large sample, it is, however, impossible to determine whether this novel strain has had, or will in the future have, any epidemiological importance.

The only Madagascan EACMV DNA-A sequence isolated during our survey was 94.7 % identical to EACMV isolates sampled in the Comoros archipelago, and corresponds to the main EACMV strain that is circulating in mainland Africa.

Amongst the 117 DNA-B components isolated in Madagascar, respectively 15 and four formed monophyletic clusters with previously sampled ACMV and EACMCV DNA-B components, whereas the 98 others were related to available DNA-B components associated with SACMV, EACMV, EACMKV and EACMZV DNA-A components, for which no clear monophyletic clustering by species could be discerned: an observation already noted in previous studies of these CMG species [32, 37] (Additional file 2: Figure S2).

Similar to the Madagascan ACMV DNA-A sequences, the 15 Madagascan ACMV DNA-B sequences were all very similar to one-another, with a mean identity of 97.1 % (all >95.4 %). The four Madagascan EACMCV DNA-B sequences were, however, considerably more diverse than both their DNA-A counterparts and the other sampled DNA-B sequences, and shared a mean identity of 91.1 % (between 84.5 and 94.8 %) with one another.

Interestingly, the 98 DNA-B components that did not obviously cluster with those of particular CMG species were quite similar, sharing a mean identity of 97.6 % (all >90.3 %; Table 1) and formed a monophyletic clade amongst all sampled DNA-B sequences. It is worth mentioning though that three of these sequences [GenBank:KJ887689, GenBank:KJ887691, GenBank:KJ887687] were more genetically distinct than the rest of the Madagascan sequences and form an outgroup within the main monophyletic Madagascan DNA-B clade. These three sequences were also more distantly related to one another than the other Madagascan DNA-B sequences in this clade.

The degree of diversity observed amongst the various Madagascan CMGs is similar to that observed amongst their counterparts on the African mainland, with the ACMV sequences being more genetically homogeneous than the EACMV-like sequences [31]. However, our study details the first ever extensive set of SACMV sequences and has revealed a surprisingly low degree of genetic diversity within this species on the island. Despite being phylogenetically closer to EACMV-like CMGs, SACMV presents with a degree of diversity that is more similar to that of ACMV.

Interestingly, our results confirm the major differences in the composition of CMG populations between Madagascar and the islands of the nearby Comoros archipelago where EACMV along with EACMKV are the dominant CMG species [32]. Despite extensive sampling, neither SACMV nor ACMV have ever been found in the Comoros.

### The geographical distribution of Madagascan CMGs

Confirming the PCR-based results, the geographical distributions of the various CMGs and their prevalence in the six main areas sampled during this study (referred to as North, West, Centre, South-west, South-east and the island of Sainte-Marie – Fig. 1) displayed some striking differences [22]. Except for Sainte-Marie, where only SACMV was isolated (*n* = 12), SACMV and ACMV were found in every sampled area. SACMV presented with a high prevalence in most of the island, except in the South-West, whereas ACMV was most prevalent in the centre of Madagascar. EACMCV was present almost everywhere (although only individual EACMCV DNA-B sequences were isolated in the West and Centre areas) albeit at a low prevalence. EACMKV was apparently restricted to the western half of Madagascar, where it occurred at a high prevalence. Finally, the only EACMV sequence sampled here was obtained from the West area.

The contrasting patterns in diversity, prevalence and geographical ranges of the different Madagascan CMG species strongly suggest that these have had distinct and complex histories of introduction to, and dissemination across, Madagascar.

### Recombination analyses

As begomovirus genomes are known to be prone to recombination [23, 24, 38], analyses to detect such events were performed to (1) reveal the evident inter-species recombination patterns within the genomes of Madagascan CMGs, (2) compare these to known recombination patterns that are evident within mainland African CMGs and (3) identify the recombinant sequences in our datasets so as to account for these during subsequent phylogeographic analyses.

**Fig. 2:**
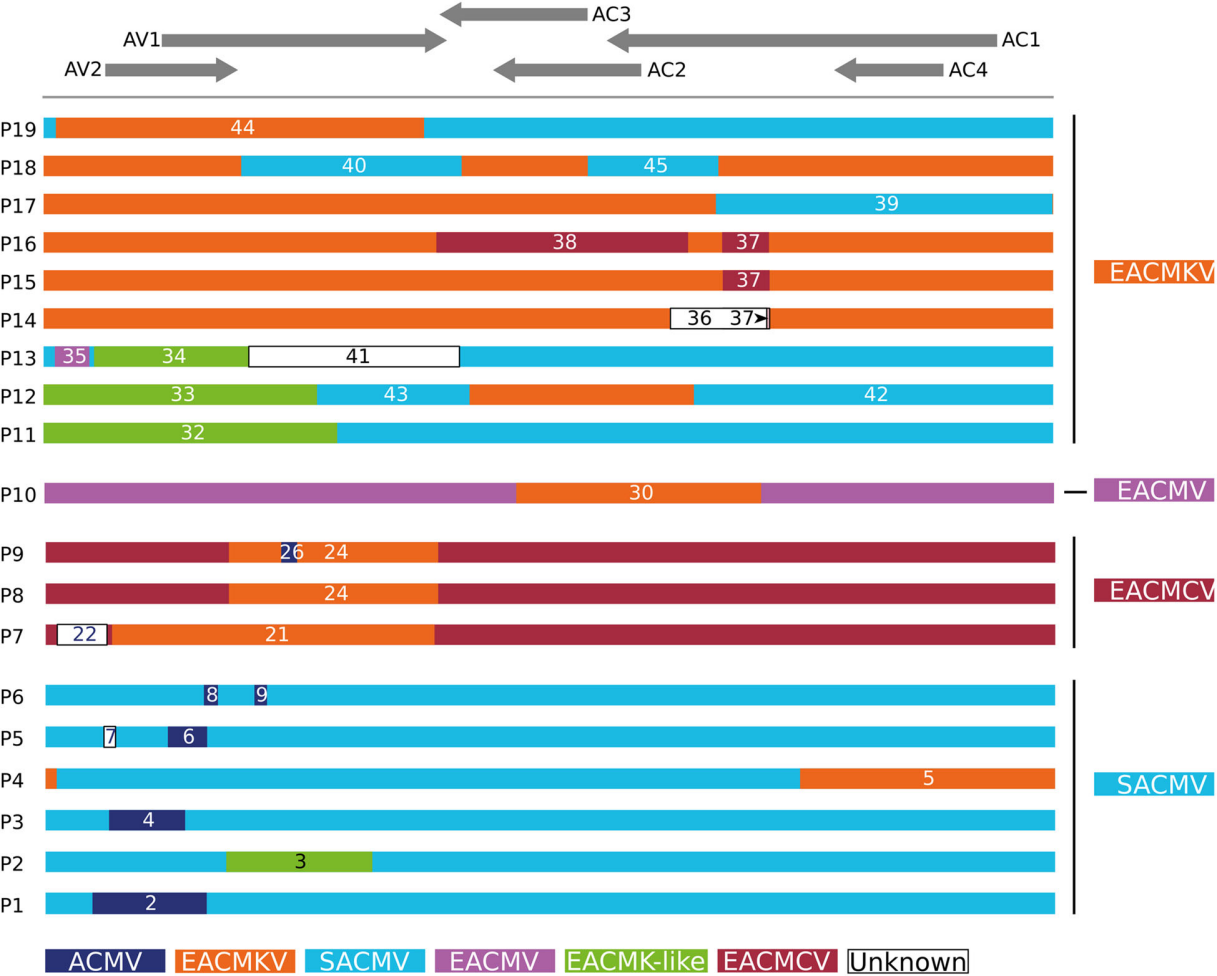
Interspecific DNA-A recombinant sequences that are found in Madagascar. Distinct profiles of recombinant DNA-A sequences isolated in Madagascar are represented with sequence portions coloured with respect to the original species they are most related to (and are therefore presumably derived from). The arrangements of DNA-A ORFs are represented at the top of the figure. Recombination events are numbered according to Table 2. The list of sequences associated with each recombination profile is available in Additional file 7: Table S2. As indicated in the text, some recombinants that are classified as EACMKV isolates according to the 89 % nucleotide identity species demarcation threshold are in fact mostly SACMV-like (i.e., SACMV was detected as the major parent)

A total of 45 inter-species recombination events were detected, 29 of which were found only in sequences sampled in Madagascar (Fig. 2, Table 2). Amongst the remaining 16 detected recombination events are those confirming the mosaic structures of many previously described CMG recombinants including: (1) EACMMV (events 10 and 11; [33]; (2) EACMZV (Event 12; [11, 33]; (3) the Kenya variant of EACMV, formally named the KE2 strain (Event 28; [13]); (4) EACMCV (Event 14; [18]); and (5) EACMV-UG (Event 27; [26]; Table 2).

**Table 2:** List of recombination events detected in CMG DNA-A and DNA-B sequences

	Event number	Recombinant	Region		Minor parent	Major parent	Methods	*P*-value
			Begin	End				
DNA-A	1	EACMMV/SACMV	1685	1974	EACMKV	Unknown	**R**GBMCST	5.5 × 10^−16^
	2	**SACMV**	131	448	ACMV	SACMV	RGBMC**S**T	4.1 × 10^−70^
	3	**SACMV**	502	907	EACMV-like	SACMV	**R**GBMCST	6.1 × 10^−60^
	4	**SACMV**	177	388	ACMV	SACMV	**R**GBMCST	3.4 × 10^−39^
	5	**SACMV**	2093	32	EACMKV	SACMV	RGBMCS**T**	1.2 × 10^−30^
	6	**SACMV**	340	449	ACMV	SACMV	RG**B**MCST	4.5 × 10^−16^
	7	**SACMV**	163	195	Unknown	SACMV	**R**GB	4.5 × 10^−06^
	8	**SACMV**	440	479	ACMV	SACMV	**R**GBS	3.6 × 10^−13^
	9	**SACMV**	580	615	ACMV	SACMV	R**G**B	4.8 × 10^−10^
	10	EACMMV	1996	2804	EACMV	SACMV	RGBMC**S**T	2.2 × 10^−29^
	11	EACMMV	54	1052	Unknown	EACMV	RGBMC**S**	5.0 × 10^−30^
	12	EACMZV	93	1924	EACMV-like	Unknown	RGBMC**S**t	2.5 × 10^−67^
	13	EACMZV	^a^1928	2077	Unknown	EACMZV	RG**B**cST	3.6 × 10^−10^
	14	EACMCV	1131	1790	Unknown	EACMV-like	**R**GBMCST	7.8 × 10^−14^
	15	EACMCV	1836	2800	EACMV	Unknown	RGBMc**S**t	1.4 × 10^−39^
	16	EACMCV	543	1103	EACMV-like	EACMCV	RG**B**MCST	2.4 × 10^−42^
	17	EACMCV	623	669	ACMV	EACMCV	RG**B**S	3.8 × 10^−12^
	18	EACMCV	1847	^a^1968	EACMV	EACMCV	RG**B**ST	5.7 × 10^−06^
	19	EACMCV	1468	1505	ACMV	EACMCV	**R**Gs	3.4 × 10^−03^
	20	EACMCV	1910	2061	EACMV	EACMCV	**R**GBS	2.0 × 10^−05^
	21	**EACMCV**	185	1079	EACMKV	EACMCV	RG**B**MCST	1.7 × 10^−25^
	22	**EACMCV**	33	^a^171	Unknown	EACMCV	R**G**BMCST	1.3 × 10^−24^
	23	EACMCV	10	1054	EACMV-like	EACMCV	RG**B**MCST	3.6 × 10^−21^
	24	**EACMCV**	509	1089	EACMKV	EACMCV	RG**B**MCST	4.0 × 10^−26^
	25	EACMCV	1835	42	EACMV	EACMCV	RGB**M**CST	1.3 × 10^−11^
	26	**EACMCV**	654	698	ACMV	EACMCV	RG**B**sT	1.9 × 10^−05^
	27	EACMV-UG	549	1007	ACMV	EACMV	RGBMC**S**T	1.2 × 10^−62^
	28	EACMV	^a^1710	2084	EACMZV	EACMV	RG**B**MCST	1.3 × 10^−22^
	29	EACMV	1680	1902	SACMV	EACMV	rgbMCST	8.9 × 10^−04^
	30	**EACMV**	1309	1988	EACMKV	EACMV	**R**GBMCST	3.0 × 10^−12^
	31	EACMV	1956	2798	EACMV	EACMKV	RGBMC**S**T	1.2 × 10^−31^
	32	**EACMKV**	1	815	EACMV-like	SACMV	**R**GBMCST	4.0 × 10^−45^
	33	**EACMKV**	1	759	EACMV-like	SACMV	RGBMCS**T**	2.9 × 10^−56^
	34	**EACMKV**	141	570	EACMV-like	SACMV	RGBMCS**T**	6.3 × 10^−59^
	35	**EACMKV**	32	^a^128	EACMV	SACMV	R**G**BST	2.4 × 10^−10^
	36	**EACMKV**	1739	2006	Unknown	Unknown	**R**BM	8.2 × 10^−04^
	37	**EACMKV**	1884	2014	EACMCV	EACMKV	RG**B**MCST	9.1 × 10^−19^
	38	**EACMKV**	1090	1788	EACMCV	EACMKV	RGBMCS**T**	9.9 × 10^−78^
	39	**EACMKV**	1865	2798	SACMV	EACMKV	RGBMCS**T**	4.6 × 10^−72^
	40	**EACMKV**	549	1160	SACMV	EACMKV	RGBMCS**T**	7.9 × 10^−46^
	41	**EACMKV**	570	1154	Unknown	EACMKV	rGB**M**C	1.7 × 10^−15^
	42	**EACMKV**	1804	2796	SACMV	EACMKV	RGBMC**S**T	1.0 × 10^−43^
	43	**EACMKV**	759	^a^1182	SACMV	EACMKV	RGBMCS**T**	1.9 × 10^−21^
	44	**EACMKV**	35	1056	EACMKV	SACMV	RGBMC**S**T	4.6 × 10^−35^
	45	**EACMKV**	1510	^a^1872	SACMV	EACMKV	R**G**BMCST	4.4 × 10^−14^
DNA-B	1	**EACMCV**	2541	53	EACMV-like	EACMCV	**R**GBMCST	7.6 × 10^−35^
	2	**EACMV-like**	1124	1461	EACMCV	EACMV-like	RGBMC**S**T	1.2 × 10^−49^
	3	**CMMGV**	1569	2697	Unknown	EACMV-like	RGBMCS**T**	5.0 × 10^−66^
	4	**EACMV-like**	2227	2287	Unknown	EACMV-like	**R**GBMCS	9.7 × 10^−23^
	5	SLCMV	2595	2712	Unknown	SLCMV	RG**B**MCST	6.7 × 10^−17^
	6	EACMV-like	2668	2780	EACMCV	EACMV-like	R**G**BMCST	4.3 × 10^−16^
	7	EACMCV	1495	2585	EACMV-like	Unkown	**R**GBmC	1.4 × 10^−07^
	8	**EACMV-like**	854	1120	Unknown	EACMV-like	**R**GBMCST	3.1 × 10^−09^
	9	EACMV-like	2345	2756	Unknown	EACMV-like	RGBMC**S**T	1.9 × 10^−15^
	10	EACMV-like	2119	2752	EACMV-like	EACMV-like	RBGMC**S**	2.9 × 10^−16^
	11	**EACMV-like**	2638	2699	Unknown	EACMV-like	**R**GBS	3.0 × 10^−05^
	12	EACMV-like	869	1597	EACMV-like	EACMV-like	rM**C**sT	8.1 × 10^−04^

For each event, the species of the recombinants and inferred parents, the recombinant region breakpoints and the list of methods which detected the event are indicated (R: RDP; G: GENECONV; B: BOOTSCAN; M: MAXCHI; C: CHIMAERA; S: SISCAN; T: 3SEQ). The reported *p*-values are for the methods in bold type and are the smallest *p*-values calculated for the region in question. Whereas upper-case letters imply that a method detected recombination with a multiple comparison corrected *p*-value <0.05, lower-case letters imply that the method detected recombination with a multiple comparison corrected *p*-value >0.05

^a^Breakpoints not inferred by RDP

Importantly, a significant number of recombination signals detected in our analyses could not be unambiguously interpreted. For example, whereas AV1 ORFs (encoding the capsid protein – CP) of EACMCV, EACMKV and EACMV are all very closely related, the AC1 ORF (encoding the replication-associated protein – Rep) of EACMKV clusters phylogenetically much more closely with that of SACMV, and the AC2 and AC3 ORFs of SACMV, EACMV and EACMKV all cluster together. While recombination is clearly the cause of discrepancies in the phylogenetic clustering patterns of these different viruses when different genome regions are considered in isolation, it is difficult to determine which species are recombinant and which are parental without the availability of additional (preferably non-recombinant) CMG DNA-A sequences.

In relation to sequences isolated in Madagascar, eight of the 130 SACMV sequences, ten of the 13 EACMCV sequences, and 17 of the 43 EACMKV sequences were detectably inter-species recombinants. The high frequency of mixed CMG infections in Madagascar, as revealed by Harimalala et al. [22], could in part explain the large numbers of inter-species recombinant sequences that we have detected here. This is especially the case for EACMV-like viruses, which have only rarely been found in single infections either on the island [22], or on mainland Africa [30].

Five of the eight recombinant SACMV sequences possess fragments of ACMV sequence located in their AV2/AV1 ORFs (Profiles P1, P3, P5 and P6; Fig. 2, Additional file 3: Table S1). Two sequences (Profile P2) have core capsid protein (CP) encoding sequences derived from an EACMV-like parental virus. The final SACMV recombinant (Profile P4) presents with an EACMKV-like sequence spanning the middle of the AC1 ORF to the virion strand origin of replication, including the whole AC4 ORF and the common region (CR). The only EACMV sequence isolated in Madagascar is a recombinant (Profile P10) with EACMKV-derived sequence spanning the AC3/AC2/AC1 ORFs. Interestingly, most of the EACMCV sequences isolated in Madagascar (10 of 13 sequences) possess a large part of their CP that is apparently derived from an EACMKV parent (Profiles P7, P8 and P9).

The recombinant EACMKV sequences display more complex recombination patterns than the other CMGs with most of the detected recombination events involving a range of either SACMV- (Profiles P11, P12, P13, P17, P18 and P19), or EACMCV-derived (Profiles P15 and P16) genome segments. Isolate MG652A11 [GenBank:KJ888083] (Profile P16), which represents a new strain of EACMKV, is characterized by its full AC3, AC2 and C-terminal AC1 ORFs being of the EACMCV type. The remaining EACMKV recombinants (Profiles P13 and P14) have sequence segments derived from a currently unsampled *Begomovirus* species. Moreover, for some recombinants (Profiles P11, P12, P13 and P19), while most of their genomes (i.e., the parts derived from their major parents) are SACMV-like, their full genome sequences are >91 % identical to EACMKV and are therefore classified as belonging to this species rather than to SACMV. This paradox can be explained by the high degrees of genetic similarity shared between SACMV and EACMKV on the complementary sense gene encoding genome regions relative to the CP encoding genome region such that the acquisition of a EACMV-like CP sequence by a SACMV variant would result in the variant being genetically more similar to EACMKV isolates than to SACMV isolates.

Since large parts of the DNA-B component sequences could not be reliably aligned, in many cases it was not possible to use phylogenies to accurately infer either which of the DNA-B sequences were recombinants, or precisely which genome fragments were transferred. Nevertheless, twelve well-supported recombination events were detected, with four of these being detectable only in DNA-B sequences sampled in Madagascar (events 1, 3, 4 and 11; Table 2).

As a whole, these results are broadly consistent with patterns of begomovirus recombination seen in previous studies. For example, just as we have found here, it has previously been shown that whereas during recombination ACMV genomes are more commonly donors (6 events in the Madagascan CMGs) than they are recipients (0 events in the Madagascan CMGs) of genome fragments (where recipients and donors are defined as the parental viruses respectively contributing the higher and lower number of nucleotides to the recombinant) [31], EACMV-like viruses are extremely prone to recombination acting as both donors (12 events in the Madagascan CMGs) and recipients (15 events in the Madagascan CMGs; [31, 33]; Fig. 2). We have additionally shown here that SACMV displays intermediate patterns of recombination relative to EACMKV and ACMV (6 events as a donor and 12 as a recipient in the Madagascan CMGs): despite its wide geographical distribution and high prevalence, we found a relatively low number of recombinant sequences, even considering the “mostly SACMV” EACMKV recombinants.

**Fig. 3:**
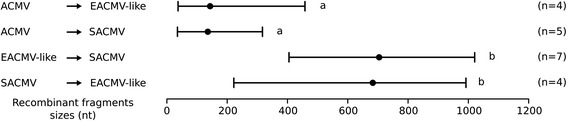
Recombinationally derived fragment sizes differ between the different CMG groups. Ranges of recombinationally derived fragment sizes are indicated for the ACMV, SACMV and EACMV-like virus lineages, with the average sizes represented by *black dots*. *Arrows* point from the inferred minor parent to the major parent. These values are based on inferred recombination events for which both parents and breakpoints were determined by the RDP4 analysis (see Table 2). The number of events corresponding to each modality is indicated, and significant differences are represented by different letters above the ranges

To confirm these patterns, we performed a recombination fragment size analysis. It appears that when fragments of ACMV origin are identified in other CMGs species, they tend to be shorter than the average recombinant fragment sizes of other CMGs (Tukey HSD test *p*-value <5 × 10^−3^, Fig. 3).

### Spatial genetic structure of CMGs in Madagascar: two contrasting situations

We performed an analysis of the population genetic structure of the Madagascan CMG clades for which we had obtained more than one sequence, specifically focusing on sequences of the core CP region which fell within a recombination cold-spot, so as to avoid the potentially confounding effects of recombination on these analyses. As a consequence of focusing on the core CP region, EACMV, EACMKV and EACMCV were merged into an EACMV-like dataset due to their close relationship in this particular part of their genomes. Five EACMKV core CP sequences that were derived from other non-CMG species by recombination (Profiles P11, P12, P13 and P18; Fig. 2, Additional file 3: Table S1) were excluded from the EACMV-like dataset. Two additional datasets corresponding to ACMV and SACMV core CP sequences were also analysed.

With the exception of a group of three ACMV sequences located in the north of Madagascar [GenBank:KJ888080, GenBank:KJ888082, GenBank:KJ888086], analysis of the core CP region of the Madagascan ACMV and SACMV isolates yielded no evidence of genetic or spatial population structure (results not shown), a result that was likely at least partially attributable to the extremely low degrees of genetic diversity observed amongst the isolates of these species.

**Fig. 4:**
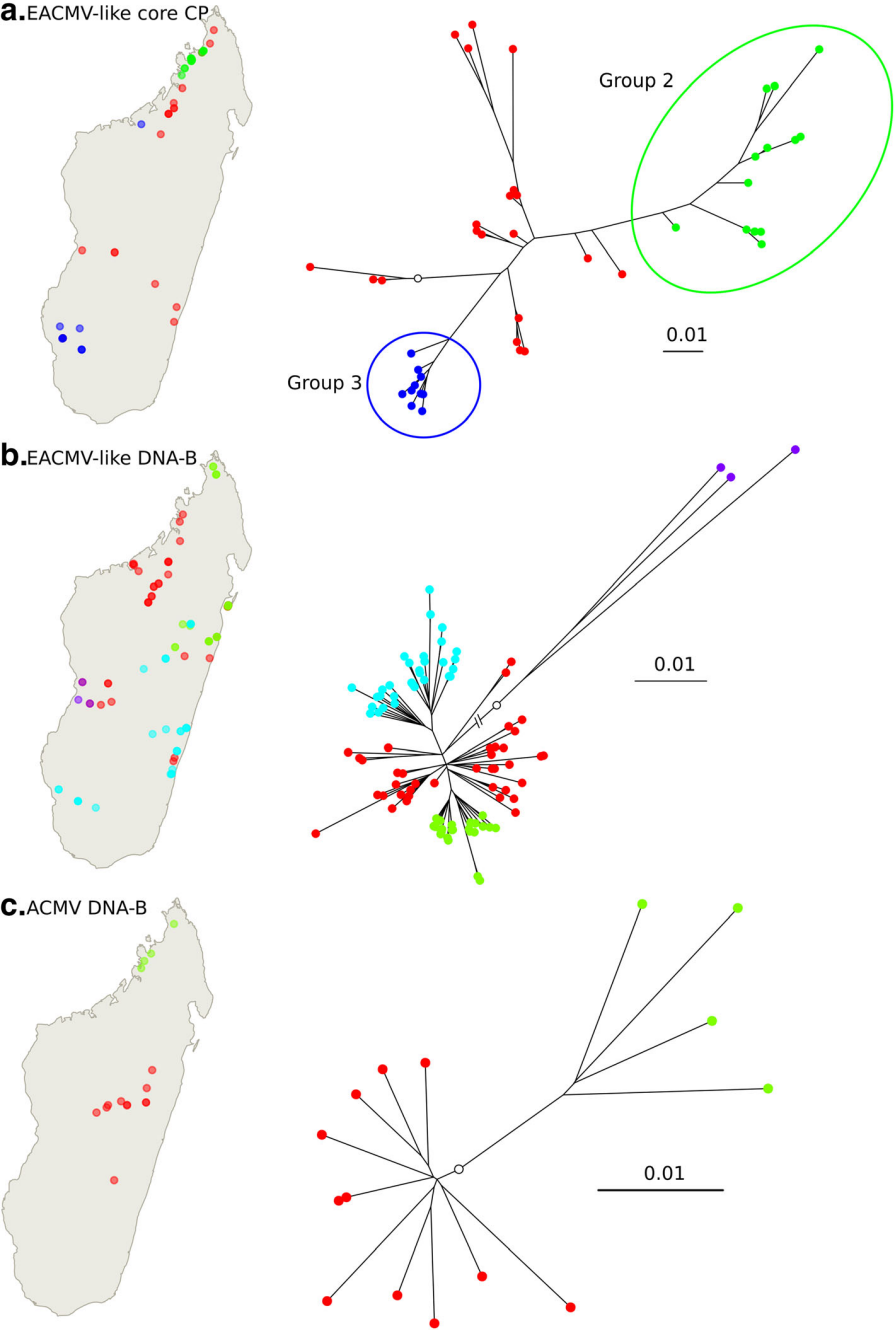
Population genetic structure revealed through discriminant analyses of principal components. Geographical location (*on the left*) and unrooted maximum-likelihood phylogenetic tree (*on the right*) of Madagascan EACMV-like DNA-A core CP (**a**), EACMV-like DNA-B (**b**) and ACMV DNA-B (**c**) sequences. Samples and tree tips are coloured according to the groups inferred in the respective DAPC analyses. *White dots* on the trees represent the location of the root on the corresponding rooted phylogeny. Note that on the tree from panel **b**, one of the branches has a reduced length for convenience of presentation

**Fig. 5:**
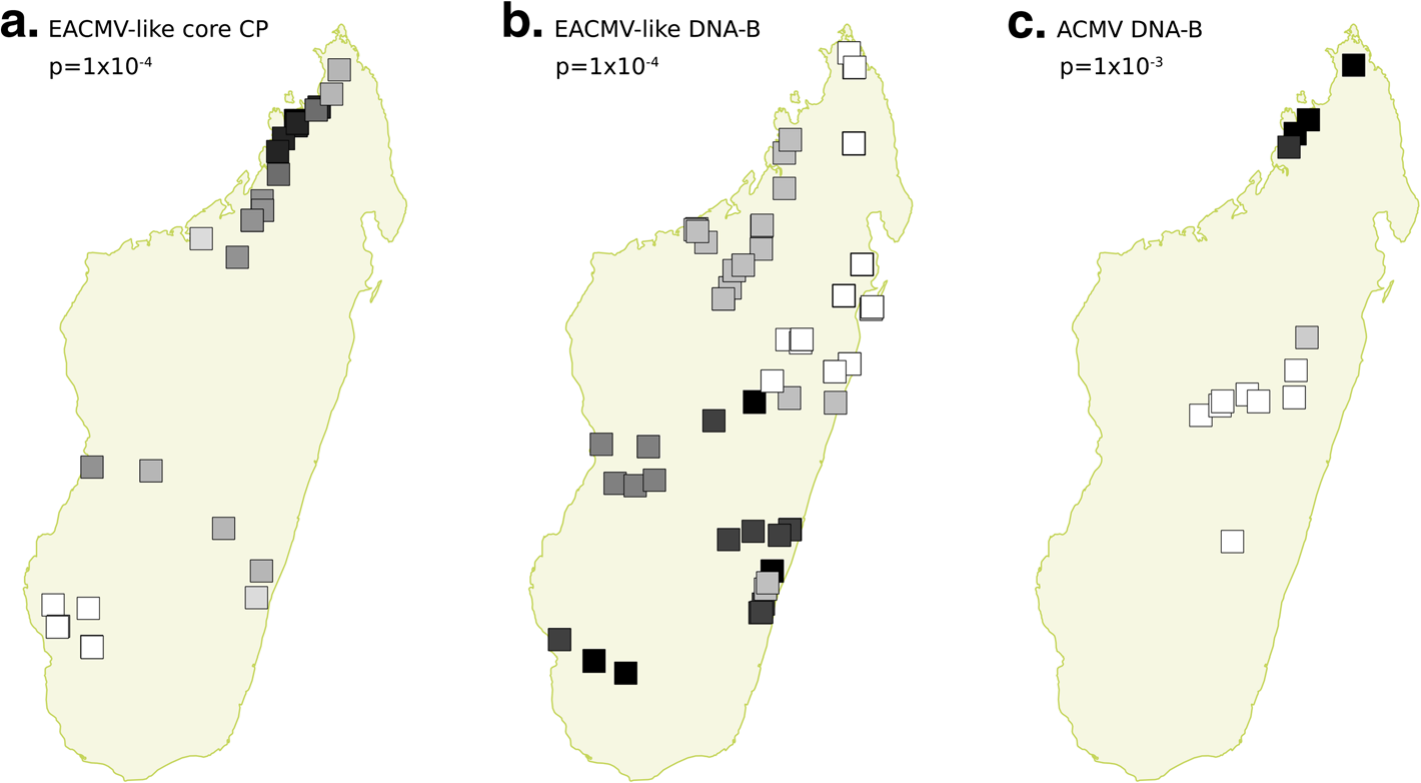
Spatial population structure revealed through spatial principal components analyses. Spatial Principal Components Analyses of the Madagascan EACMV-like DNA-A core CP (**a**), EACMV-like DNA-B (**b**) and ACMV DNA-B (**c**) sequences. The locations of samples are represented with *squares* coloured according to a *black* to *white* gradient corresponding to the eigenvalues for the first axis of the sPCA. The *p*-values of the global spatial structure detected in each dataset are presented

Our analysis of the genetic structure of the EACMV-like core CP sequences (Fig. 4a) revealed the existence of two monophyletic groups (Groups 2 and 3, respectively in green corresponding to EACMKV and EACMCV isolates and blue corresponding to EACMKV isolates) and one basal group (Group 1, in red) corresponding to EACMKV, EACMCV and EACMV isolates that could not obviously be discriminated by the Discriminant Analysis of Principal Components (DAPC) method. When visualising the geographical distribution of each group, it is clear that group 2 is restricted to a small area in the north, while group 3 is located mostly in the south-west with only one isolate in the north-west, suggesting that at least two sub-populations (groups 2 and 3) of EACMV-like viruses are present in Madagascar. This geographical structuring was confirmed by the spatial Principal Components Analysis (sPCA; *p*-value = 1 × 10^−4^, Fig. 5a). The first axis of the analysis, which represents 27 % of the variance and a large positive autocorrelation index (Moran’s I), permits clear separation of the three distinct groups inferred by the DAPC analysis. Additionally, when exploring the spatial structure of group 1 alone, a significant pattern of isolation by distance across a North/South gradient was detected (*p*-value = 5 × 10^−3^; data not shown).

The genetic structure of Madagascan CMG DNA-B-components was investigated using their full sequences, for two datasets corresponding respectively to the 15 ACMV DNA-B components and the 98 monophyletic Madagascan EACMV-like isolates referred to as the EACMV-like DNA-B component dataset.

Contrasting with the absence of structure that was evident for the core CP sequences of ACMV DNA-A components, the DAPC analysis performed on the 15 ACMV DNA-B components clearly distinguished sequences isolated in the North from those isolated in the centre of Madagascar (Fig. 4c). This spatial clustering was confirmed with the sPCA analysis (*p*-value = 1 × 10^−3^; Fig. 5c).

For EACMV-like DNA-B components, the DAPC analysis detected four clusters (Fig. 4b). One cluster of three isolates [GenBank:KJ887689, GenBank:KJ887691, GenBank:KJ887687] (represented in purple in Fig. 4b) corresponds to the three divergent EAMKV DNA-B sequences, all of which were sampled in the western parts of Madagascar. Interestingly, when we excluded these sequences from the dataset, the sPCA analysis indicated a pattern of isolation by distance (*p*-value = 1 × 10^−4^) with a north/south spatial gradient (Fig. 5b) – a finding consistent with our analysis of the core CP sequences.

The patterns of isolation by distance detected in the EACMV-like DNA-A and DNA-B datasets and the ACMV DNA-B dataset suggests the occurrence of limitations on the long distance dissemination of viruses in Madagascar: a process which presumably must involve either transmission by *B. tabaci* or human mediated transportation of cuttings. Given the available data and the absence of any profound differentiation between mainland African and SWIO CMG populations, it is also plausible that multiple and recent independent introductions of distinct DNA-A and DNA-B variants of these viruses to different parts of the island may have contributed to the observed spatial structure.

It is, however, not possible from these analyses to definitively determine why the Madagascan ACMV and SACMV DNA-A populations lack any discernible spatial or genetic structure. Two equally plausible explanations may be: (1) that these viruses only recently arrived in Madagascar and that the same DNA-A variants of these viruses were rapidly disseminated across the cassava growing regions of the island; or (2) that single highly fit variants of these DNA-A components have recently arisen in Madagascar, and that these have spread throughout the island during a selective sweep that purged all but the descendants of these variants.

### The history of CMG introductions to the SWIO islands

Reconstructing the movement histories of CMGs is a challenging task because factors such as the density of sampling, the range of sampling dates, or recombination events can all profoundly affect time-scaled phylogenetics-based inferences of past virus dissemination events.

Nonetheless, to investigate the movement histories of ACMV and EACMV-like viruses between mainland Africa and the SWIO islands so as to infer when, and from where these viruses were introduced into Madagascar, we undertook a Bayesian probabilistic phylogeographic analysis of the available core CP sequences of these CMGs. A similar analysis for the SACMV sequences could not be performed due to a lack of available sequence data from mainland Africa.

As expected considering the narrow duration over which the analysed CMG isolates were sampled (between 1990 and 2012 for ACMV DNA-A components, with 90 % of the sequences having been sampled between 2006 and 2012; between 1996 and 2011 for EACMV-like DNA-A components, with 95 % of the sequences having been sampled between 2001 and 2011; 1982–2012 for ACMV DNA-B components, with 90 % of sequences having been sampled between 2006 and 2012; 1997–2011 for EACMV-like B components, with 95 % of sequences having been sampled between 2000 and 2011), the Path-O-Gen analyses revealed that our datasets lacked evidence of strong temporal signals (Table 1). Nonetheless, when sampling dates were randomly shuffled between samples, the evolutionary rates inferred by the BEAST analyses were substantially lower than those inferred from the real data (Additional file 4: Figure S3), suggesting that although the temporal signal in the data is not strong, it remains present. As a whole, however, these results indicated that all dates and evolutionary rates inferred during the subsequent phylogeographic analyses should be treated with caution.

Using the spatial coordinates of our samples, we defined several discrete geographic sampling locations for each dataset (respectively seven and eight for the ACMV and EACMV-like datasets). By associating one of these discrete sampling location states to each analysed sequence, and accounting for sampling dates, the “discrete” phylogeographic analysis that we performed permitted us to identify when and where (amongst the seven or eight defined sampling locations) ancestral CMG sequences (represented by internal nodes on phylogenetic trees in the posterior distribution of trees) probably existed.

**Fig. 6:**
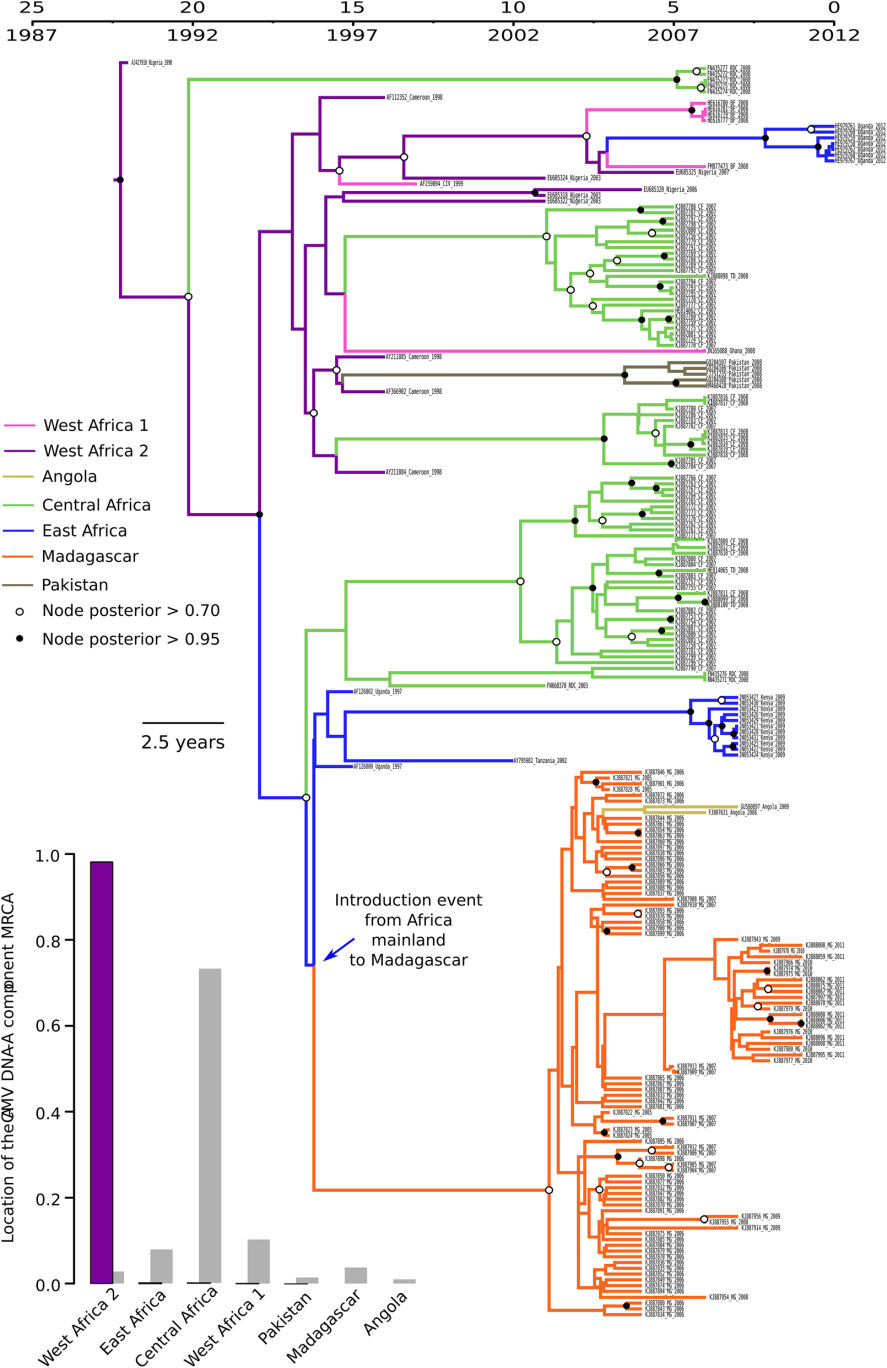
Maximum clade credibility tree constructed from the ACMV core CP dataset. Branches are coloured according to the most probable location state of the node on their right (i.e., the likely geographical location of the ancestral sequence represented by this node). The time-scale of evolutionary changes represented in the tree is indicated by the scale bar above it. Whereas *filled circles* that are associated with nodes indicate >95 % posterior probability support for the branches to their left, *open circles* indicate nodes with >70 % posterior support for these branches. Nodes to the right of branches with <70 % support are left unlabelled. The bar graph indicates location probabilities of the node at the root of the tree (i.e., the most recent common ancestor of all the sequences represented in the tree). *Grey bars* represent the probabilities obtained with randomization of the tip locations. The probable introduction event from Africa to Madagascar is indicated with a *blue arrow*

Confirming the results obtained from the diversity and population genetic analyses, the discrete phylogeographic analysis performed on the ACMV core CP sequences suggested that all of the Madagascan ACMV sequences had likely descended from a single sequence introduced to the island from East Africa (location probability = 0.93; Fig. 6). This inferred geographical origin of the Madagascan ACMV DNA-A sequences should, however, be interpreted with caution as the posterior probability of the branch immediately predating the Madagascan ACMV group is low, suggesting that the precise branching location of this clade within the broader mainland African ACMV clade has not been fully resolved. Consistent with the low degrees of diversity observed amongst the Madagascan ACMV core CP sequences, these analyses also indicated that the migration event likely occurred very recently - between 1996 and 2003 (95 % HPD ranging from 1995 to 2004).

Surprisingly, two ACMV sequences sampled in Angola are inferred to be descendants of a Madagascan ACMV lineage, with our analysis yielding very strong support (BF = 228) for movements between these locations. This is the first reported example of a CMG moving from a SWIO island back onto the African continent. Crucially, our finding that the Madagascan ACMV population was founded by an isolate transferred to the island from Africa was also supported by our phylogeographic analysis of the ACMV DNA-B components (Additional file 5: Figure S4).

**Fig. 7:**
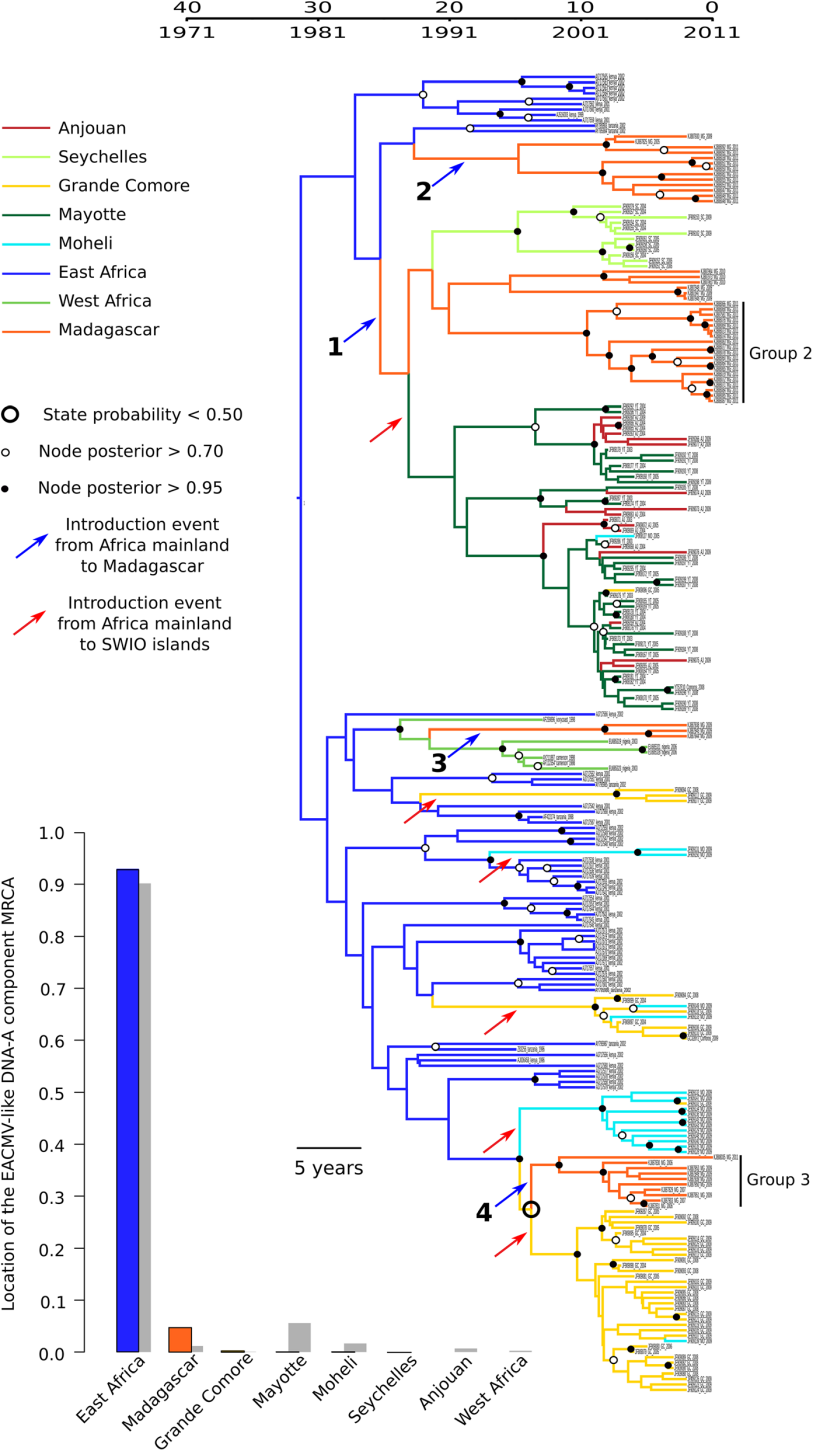
Maximum clade credibility tree constructed from the EACMV-like core CP dataset. Branches are coloured according to the most probable location state of the node on their right (i.e., the likely geographical location of the ancestral sequence represented by this node). The *large black circle* around one of the nodes indicates that the state probability at this node is less than 0.5 (i.e., there is less than 50 % confidence in the indicated location being the actual place where this ancestral sequence existed). The time-scale of evolutionary changes represented in the tree is indicated by the scale bar above it. Whereas *filled circles* that are associated with nodes indicate >95 % posterior probability support for the branches to their left, *open circles* indicate nodes with >70 % posterior support for these branches. Nodes to the right of branches with <70 % support are left unlabelled. The bar graph indicates location probabilities of the node at the root of the tree (i.e., the most recent common ancestor of all the sequences represented in the tree). *Grey bars* represent the probabilities obtained with randomization of the tip locations. Probable introduction events from Africa to the SWIO islands are indicated with *red arrows*, while introduction events from Africa to Madagascar are numbered and indicated by *blue arrows*. Groups 2 and 3 inferred in the DAPC analysis of the EACMV-like core CP sequences are indicated on the tree

Our EACMV-like core CP phylogeographic analysis inferred that there had been at least five independent introduction events of these CMGs into the Comoros islands from mainland Africa (Fig. 7): a finding which confirms the results of a previous study [32]. Our results additionally infer that the presence of EACMV-like viruses in Madagascar is the consequence of three to four independent introduction events. For two of these events, due to branches with low support, it is unclear whether sequences from Madagascar and the SWIO islands are directly related or not. Nonetheless, the Bayesian stochastic search variable selection (BSSVS) analysis strongly support the epidemiological linkage inferred between East Africa and Madagascar (BF = 91.6) but not between Madagascar and any of the other SWIO islands. These findings support the hypothesis that virus movements between Madagascar and the Comoros are far more constrained than those between Mainland Africa and these islands.

The first two events (events 1 and 2 on Fig. 7) are probable introduction from East Africa respectively between 1988 and 1990 (HPD ranging from 1982 to 1997) and 1988 and 1996 (HPD ranging from 1983 to 2003). These two events are difficult to interpret, as they are obscured by poorly resolved branches in the underlying phylogenetic tree topology, but the corresponding core CP sequences (derived from EACMV, EACMKV and EACMCV isolates) are closely related to the core CP sequences of EACMV-like isolates (i.e., EACMV and EACMKV) introduced to Mayotte at approximately the same time. Interestingly, amongst the isolates from Madagascar, we found a monophyletic group of EACMV-like core CP sequences belonging to EACMKV and EACMCV isolates sampled in the north of the country (group 2 of EACMV-like core CP sequences in the DAPC analysis; Fig. 4). This clade, containing some recombinant sequences between EACMKV and EACMCV, as well as the new Madagascan strain of EACMKV, clusters with other Madagascan isolates and could not be associated with an independent introduction event in our phylogeographic analysis. Moreover, the mean substitution rate inferred for this particular group is significantly different to the rest of the tree (Wilcoxon test, *p*-value = 2.75 × 10^−3^). Even if the interpretation of these rates is not straightforward due to the many biases exposed earlier, these results tend to support a hypothesis involving the *in situ* diversification of EACMV-like virus population over a hypothesis involving large numbers of independent introductions of distinct EACMV-like virus variants.

The third EACMV-like virus introduction event (event 3 on Fig. 7) likely involved a non-recombinant lineage of EACMCV sequences transferred onto the island between 1984 and 2003 (HPD ranging from 1971 to 2006). The last event (event 4 on Fig. 7) involved the transfer onto the island between 1997 and 1999 (95 % HPD ranging from 1994 to 2003) of the non-recombinant EACMKV lineage which is mostly located in the South-West (corresponding to group 3 of EACMV-like core CP sequences in the DAPC analysis; Fig. 4). Individual variants within this particular EACMKV clade may subsequently have been concomitantly introduced to both Madagascar and Grande Comore.

Based on their monophyletic clustering, our phylogeographic analysis of the EACMV-like DNA-B component sequences suggests that the Madagascan sequences in this group originate from a single introduction event from East Africa (Additional file 6: Figure S5).

It is noteworthy that despite the Madagascan EACMV-like DNA-B sequences being most similar to the EACMV-like DNA-B sequences circulating in the Comoros archipelago, our phylogeographic analyses failed to yield any evidence of EACMV-like virus movements between the two islands. It is possible that either the same or closely related variants were simultaneously introduced to both Madagascar and the Comoros archipelago, after which the distinct populations evolved in isolation along independent trajectories.

## Discussion

The co-occurrence in Madagascar of six CMG species within the same geographical area is a major opportunity to study the evolutionary, spatial and temporal dynamics of several distinct virus species concurrently evolving within the same ecological context. Towards this end we constructed viral sequence datasets representing the four most prevalent Madagascan CMG species (SACMV, ACMV, EACMKV and EACMCV) and compared their population structures and movement dynamics within the broader context of CMGs found elsewhere on the SWIO islands and on mainland Africa. We show both that despite sharing the same host and vector species, these four CMG species have distinct patterns of recombination and varying population structures, and that they have likely all been independently introduced to Madagascar.

We confirm previously described differences between recombination patterns in ACMV and other CMG species. Specifically, whereas ACMV is apparently an avid donor of small sequence fragments during inter-species recombination events, it has only very rarely been inferred to be a recipient of foreign sequence fragments of any size: a pattern that contrasts starkly with that of other CMG species which frequently exchange genomic fragments of widely varying sizes with one another. The only example of an inter-species recombination event involving an ACMV genome as the recipient of a sequence fragment derived from some other virus species (i.e., a recombination event with ACMV as the major parent) is that observed in the recently discovered CMG species, ACMBFV. This species is effectively a recombinant between a West African ACMV variant and a presently unsampled, but probably monopartite begomovirus species closely related to *Tomato leaf curl Cameroon virus* [14].

Besides the lower frequencies of productive genetic transfers between ACMV and other CMGs, we show here that in recombinant SACMV and EACMV-like viruses (i.e., EACMV, EACMKV and EACMCV) presenting with ACMV derived sequence tracts, the lengths of these tracts are, on average, significantly shorter than those which are detectably transferred between other CMGs. While instances are known of large genome fragments having been transferred between ACMV and other CMGs – for example the largely ACMV-derived coat protein gene that characterizes the EACMV-UG strain [26] – it is plausible that ACMV genome sequences are largely “incompatible” with those of other CMGs. A crucial factor may be that ACMV is the most divergent of the African CMGs. It is well established that the degree of compatibility of a genome region within the context of a foreign genetic background is closely associated with the degree to which it differs from the genome region that it replaces: i.e., the likelihood of negative epistatic interactions between the foreign fragment and the genomic background within which it finds itself increases with greater degrees of evolutionary divergence between the genomic background from that in which the fragment evolved [39–41].

We have revealed that Madagascar is apparently relatively isolated epidemiologically from the nearby SWIO islands of the Comoros and Seychelles. Besides these islands displaying striking differences in the species compositions of their CMG populations, our phylogeographic analyses failed to reveal any statistical support for evidence of CMG movements between Madagascar and other SWIO islands. Instead it is apparent that EACMV-like variants have been directly transferred at least three times from Africa to Madagascar and that they have likely been circulating on this island for approximately the same time-period as those found on the Comoros and Seychelles archipelagos.

Although our datasets were inadequate to provide a detailed description of the temporal movement dynamics of CMGs within Madagascar, the contrasting diversity and spatial dynamics of different CMG species on the island indicated that these species might display markedly different histories of introduction to the island. Specifically, whereas EACMV-like viruses appear to have been introduced multiple times to the islands, the two most prevalent CMGs on the island, ACMV and SACMV, both appear to have been introduced to the island only once in the very recent past.

Whereas no spatial structure was evident for the genetically homogeneous Madagascan ACMV and SACMV populations, the EACMV-like virus population, which consists of three distinct species, has probably been present on the island for longer than both ACMV and SACMV and displays a degree of spatial structure with discernible genetic differentiation along a north/south axis. This spatial differentiation could be the result of the short-range dissemination of the viruses by local dispersal either through human mediated transport of infected cuttings or through *B. tabaci* mediated transmission. The fact that similar genetic imprints were not detectable in the SACMV and ACMV populations might be due to these species disseminating more rapidly than EACMV-like viruses. Specifically if dissemination occurs at a faster pace than the rate at which genetic polymorphisms accumulate within a virus population, there will be no discernible genetic imprint permitting the precise tracking of past movements. It remains to be determined, however, whether the widespread distribution across Madagascar of genetically homogenous ACMV and SACMV populations is associated with either more frequent longer-distance human or *B. tabaci* mediated transport of these species compared to the EACMV-like viruses.

## Conclusion

The results of this study highlight the complexity of CMD in Madagascar, with each CMG species having their own epidemiological and evolutionary dynamics despite sharing the same ecological niche. Whereas ACMV and SACMV dominate in terms of prevalence and spread, respectively in the high and low altitude regions [22], the multiple introduction events of EACMV-like viruses as well as their tendency to recombine frequently and to be involved in mixed infections [22], taken together suggests a high degree of modularity within this group of viruses. It is plausible that these factors may increase the risk of recombinant EACMV-like viruses with altered pathogenic properties emerging as an additional threat to cassava cultivation in Madagascar.

Moreover, our analyses suggested evidence of at least one CMG movement from Madagascar to the region around Angola on the African mainland. This finding dramatically increases the potentially global epidemiological importance of any novel pathogenic CMG variants that might arise on the SWIO islands, and suggests that measures to reduce the movements of CMG infected cassava plants between Madagascar, the other SWIO islands and mainland Africa may be warranted. In this regard it is interesting to note that despite their geographical isolation, Madagascan cassava landraces are not at all genetically distinct from those found across mainland Africa [42]. This may be attributable to a severe CMD epidemic in Madagascar in the 1930s which caused the almost total elimination of local varieties [34]. Due to this devastation it is likely that many of the current Madagascan ‘landraces’ are in fact relatively recent introductions from mainland Africa [34]. The obvious incongruence between the date of this epidemic and our molecular and phylogeographic analyses strongly suggest that the CMG variants that are presently circulating on the island are not descendants of those that caused the epidemic of the 1930s. It would surely be interesting to determine what role the importation of cassava germplasm from Africa has had on currently observable CMG demographics - both from the perspective of CMGs being imported onto the island within infected germplasm and from the perspective of how these CMGs fare when infecting different imported and local cassava varieties.

## Methods

### Sampling, cloning and sequencing of CMG sequences from Madagascar

Cassava leaf samples displaying typical CMD symptoms were collected in Madagascar between 2005 and 2011. Spatial coordinates were recorded for each sample and leaves were dried using calcium chloride. Total plant DNA was extracted from dried leaves using the DNeasy Plant Mini Kit (QIAGEN France) according to the manufacturer’s instructions. A total of 279 complete DNA-A and 117 complete DNA-B sequences (Additional file 7: Table S2) were obtained using a previously described RFLP-RCA method [43]. Full genome amplicons were digested using the restriction enzymes *Bam*HI, *Nco*I, *Eco*RI and *Apa*I, before ligation into similarly linearized pGem-T or pGem-7zf cloning vectors (Promega, USA) and transformation into *E. coli*. The resulting clones were sequenced using primer-walking Sanger sequencing methods by a commercial company (Macrogen, Europe). Sequences were edited and assembled using the software, DNA Baser (Heracle BioSoft S.R.L., Romania). All sequences obtained in this study are available on GenBank [KJ887581:KJ888100; KM885990:KM886005] (Additional file 7: Table S2).

#### Alignments, virus classification and phylogenetic trees

All sequence alignments were produced using the MUSCLE [44] method as implemented in Geneious 6.1.7 (Biomatters Ltd, New Zealand) and MEGA 5.2 [45] with default parameters. All alignments were manually edited.

A pairwise nucleotide identity matrix was calculated for full length DNA-A sequences using SDT v1.2 [46] and was used to assign each sequence to a viral species and strain, according to the ICTV approved begomovirus species (>91 % DNA-A identity) and strain (>94 % DNA-A identity) demarcation thresholds [36].

Approximately-maximum-likelihood phylogenetic trees were constructed using the computer program FastTree (v2.1, [47]), with the GTR-CAT nucleotide substitution model and a gamma distribution of 20 substitution rates categories. Branch support was evaluated using the Shimodaira-Hasegawa-like test implemented in that program.

### Recombination analyses

Detection of potential recombinant sequences, identification of sequences closely related to parental sequences and localization of recombination breakpoints were carried out using the RDP [48], GENECONV [24], BOOTSCAN [49], MAXIMUM CHI SQUARE [50], CHIMAERA [49], SISCAN [51], and 3SEQ [52] recombination detection methods implemented in RDP4 [53].

The recombination analysis was performed on a dataset of 626 DNA-A sequences, containing 351 new CMG DNA-A sequences (279 from Madagascar and Comoros and 72 from the Central African Republic and Chad) aligned together with 114 CMG sequences recently isolated from the Comoros and Seychelles archipelagos [32], 152 representative CMG DNA-A sequences available from GenBank and 9 DNA-A-like sequences of monopartite begomoviruses from Africa and Madagascar.

Despite the high diversity of DNA-B sequences and the inherent difficulties in generating accurate alignments of these, an additional recombination analysis was performed on a dataset of 336 DNA-B sequences containing 187 new CMG sequences (117 from Madagascar and Comoros and 70 from the Central African Republic and Chad) aligned together with 55 DNA-B sequences from the Comoros and Seychelles archipelagos [32], and 94 representative CMG DNA-B sequences available from GenBank.

For the sake of both computational speed and the facilitation of further analyses, divergent CMG taxa (corresponding to ACMV, SACMV, and EACMV-like [EACMV, EACMKV and EACMCV] for DNA-A; ACMV, EACMCV, and EACMV/SACMV-like for DNA-B) were analysed separately, using the “select group” option in RDP4.

Default settings for the different detection methods and a Bonferroni corrected *p*-value cut-off of 0.05 were used. The only recombination signals that were considered to represent definitive evidence of recombination were those with associated phylogenetic support that were detectable by three or more of the seven applied recombination detection methods. The breakpoint positions and recombinant sequences inferred for each potential recombination event were manually checked and adjusted when necessary using the range of analysis cross-checking tools available in RDP4.

### Genetic and spatial structure of CMGs in Madagascar

The existence of potential genetic structure in Madagascan CMG populations was investigated using the Discriminant Analysis of Principal Components (DAPC; [54]) method implemented in the R 3.0.1 Adegenet package [55, 56]. DAPC is a multivariate method designed both to infer clusters of genetically related sequences, and to describe the variability between these sequence clusters. Firstly, a principal component analysis (PCA) of allelic diversity was performed to reduce the number of variables before using a sequential K-means clustering procedure to infer an optimal number of groups (selected using the Bayesian information criterion). Then, a first step of data transformation was performed using a second PCA on the raw data, to obtain a lower number of uncorrelated variables describing the dataset. Subsequently, a discriminant analysis (DA; [57]) was performed upon these variables in order to maximize the variation between groups (with the number of groups having been defined in the first step) while minimizing the variation within each group. This method had the advantage of not relying on a particular genetic model. Analysis of the repartition of each group with respect to the DA axes was used to yield information relating to the genetic structure of the dataset.

In addition to analysing genetic structure within the Madagascan CMG populations, we also looked for evidence of spatial structure within the distributions of these sequences across Madagascar. For this we used spatial principal components analysis (sPCA; [55]), which was also implemented in the R Adegenet package. This method relies on a modification of classical PCA to take the spatial autocorrelation of genetic variability into account. Unlike PCA, which seeks to find independent synthetic variables that maximize variance, sPCA variables are selected to maximize the product of the variance and a spatial autocorrelation index: Moran’s I. One can choose the variables which have a large associated variance and a large positive Moran’s I, therefore representing patterns of isolation by distance (distant individuals tend to be more genetically distant), or a negative Moran’s I (close individuals tend to be more genetically distant), respectively referred to in the package as global and local structures. The significance of any structure was inferred using a permutation test implemented in the package. Specifically, for each dataset we employed a Delaunay triangulation network to describe connections between our isolates, and tested the significance of inferred spatial structures using 9999 permutations.

### Phylogeography of CMGs on the South-West Indian ocean islands

The spatial and temporal dynamics of CMGs in the SWIO islands were investigated using a discrete symmetric diffusion model implemented within the Bayesian inference framework of the computer program BEAST v1.8.2 [58].

A first dataset, called EACMV-like, was assembled from 244 EACMV, EACMKV, and EACMCV sequences isolated from the Comoros (117; including the islands of Mayotte, Anjouan, Grande Comore and Moheli), Seychelles (12), Madagascar (51), and the African continent (64). Each of the sequences had a corresponding GPS coordinate and sampling date, and was assigned to one of the following eight discrete sampling locations: East Africa, West Africa, Seychelles, Mayotte, Anjouan, Grande Comore, Moheli and Madagascar.

A second dataset, called ACMV, was assembled from 218 ACMV sequences isolated from Madagascar (93), and the African continent (123). The sequences from the African continent were hierarchically clustered based on their sampling coordinates into five discrete geographic locations (West 1, West 2, Centre, East and Angola). Because samples were not available for both ACMV-like and EACMV-like viruses from all of the same geographical locations the discretization of the samples using hierarchically cluster methods were necessarily different. Two additional discrete locations corresponding to Pakistan and Madagascar were also defined.

To avoid the confounding effects of genetic recombination on phylogenetic reconstructions, only sequences corresponding to a recombination cold-spot within the core region of the capsid encoding ORF (core CP; [23, 32]) were used in the subsequent molecular clock and phylogeographic analyses. Only sequences for which the entire core CP region was representative of the relevant CMG species (i.e., viruses in the ACMV or EACMV-like datasets that had not recombinationally acquired core CP regions from other divergent *Begomovirus* species) were included in the analyses.

The GTR + I + G model was selected as the best fit nucleotide substitution model using RDP4, for all four datasets and the BEAST analyses were performed using a lognormal relaxed clock model with a Bayesian SkyGrid coalescent tree prior with 100 grid points; both of which were selected as the best-fitting models using the path sampling and stepping-stone model selection procedures [59]. Three independent runs comprising a total of either 400 or 800 million iterations of the Marko chain were performed for each dataset and where necessary, combined using LogCombiner after the removal of an appropriate burn-in to ensure that the effective sample sizes for parameters all exceeded 200. The Maximum Clade Credibility trees (MCC) was constructed using TreeAnnotator and was visualized using the computer program FigTree (available at http://tree.bio.ed.ac.uk/software/figtree/). A Bayesian stochastic search variable selection (BSSVS) approach, described in detail by Lemey et al. [60], was used to identify well-supported epidemiological links between locations using Bayes factor (BF) tests [61], with BF values >5 taken as representing significant evidence of migration between the discrete sampling locations considered.

In order to estimate biases due to differences in sampling sizes between the discrete locations, the analyses were also carried out as above but with the location states of the sequences randomized using an additional operator in the MCMC procedure. The location state probabilities of the root node determined during these analyses were compared with those determined for the datasets analysed without the location state randomization setting.

As only two African SACMV sequences exist in the databases, the history of SACMV movements between continental Africa and Madagascar could not be properly investigated using a discrete phylogeographic analysis approach.

Finally, two additional phylogeographic analyses were performed on datasets consisting of 215 full-length EACMV-like DNA-B components (corresponding to 98 Madagascan sequences, 51 form the Comoros archipelagos and 65 from mainland Africa) and 95 full-length ACMV DNA-B components (corresponding to 15 from Madagascar and 80 from mainland Africa). The same parameters and procedures used for the DNA-A analyses were applied for the DNA-B analyses except that seven discrete sampling location states were used for the EACMV-like DNA-B phylogeographic analysis (corresponding to East Africa, Central Africa, Grande Comore, Mayotte, Anjouan, Moheli and Madagascar) and four discrete sampling location states were used for the ACMV DNA-B phylogeographic analysis (corresponding to West Africa, Central Africa, East Africa and Madagascar).

Importantly, as dates and rates inferred from BEAST analyses are strongly affected by the range of the sampling dates, the temporal signal of each of our datasets was investigated using two methods. Firstly, we used the computer program Path-O-Gen 1.4 (available at http://tree.bio.ed.ac.uk/software/pathogen/) which, when provided with a phylogenetic tree, performs a linear regression between the sampling dates of the sequences and their genetic distances from the root (i.e., the point in the tree that, assuming a molecular clock model of evolution would best represent the most recent common ancestor of all the sequences used to construct the tree). A correlation coefficient close to one would indicate evidence of the corresponding sequence dataset being compatible with a strict clock evolutionary model (i.e., with equal rates of nucleotide substitution across all the branches of the phylogeny). Alternatively, a correlation coefficient between 0 and 1 would imply that the use of a relaxed clock evolutionary model is more appropriate. A negative or zero correlation coefficient would imply that the data are absolutely not clocklike and then that the genetic variability evident in the data could not be properly related to sampling times (i.e., the absence of sufficient temporal signal in the dataset to confidently infer coalescence times or substitution rates). Secondly, we performed a tip-date randomization technique previously described in other studies [29, 62]. For each dataset, ten independent randomizations of the sampling dates associated with each sequence were performed. The mean substitution rate distribution of these randomized datasets was inferred with BEAST, and then compared with the mean substitution rates inferred from the real datasets. A correspondence between rate estimates of real and randomized datasets would suggest a lack of temporal signal in our data.

## Additional files

Additional file 1: Figure S1. Geographical map of African CMGs. The nine CMG species identified in Africa and on the SWIO islands are represented by a coloured circle in the countries where they have been isolated. (TIFF 600 kb) Additional file 2: Figure S2. Phylogenies of DNA-A and DNA-B component representatives sequences of Cassava Mosaic Geminiviruses. Approximately-maximum-likelihood phylogenetic trees of DNA-A (panel A) and DNA-B (panel B) components of CMGs are represented. For the sake of clarity, only representative sequences have been included in the phylogenetic reconstruction. (TIFF 4294 kb) Additional file 3: Table S1. List of recombination profiles for Malagasy sequences. For each of the recombination profiles, the numbers of associated recombination events are indicated, as well as species and accession numbers of the corresponding recombinant sequences. (XLSX 8 kb) Additional file 4: Figure S3. Comparison of substitution rates inferred with tip-dates randomization. The substitution rates inferred by BEAST with and without randomization of the sequences sampling dates are indicated on a base-ten log scale for each dataset. Panel A: ACMV DNA-A core CP; Panel B: ACMV DNA-B; Panel C: EACMV-like DNA-A core CP; Panel D: EACMV-like DNA-B. Vertical lines represent the 95 % highest posterior density (HPD). Real datasets values and randomized datasets values are coloured respectively in red and black. (TIFF 349 kb) Additional file 5: Figure S4. Maximum clade credibility tree constructed from the ACMV DNA-B dataset. Branches are coloured according to the most probable location state of the node on their right (i.e., the likely geographical location of the ancestral sequence represented by this node). The time-scale of evolutionary changes represented in the tree is indicated by the scale bar above it. Whereas filled circles that are associated with nodes indicate >95 % posterior probability support for the branches to their left, open circles indicate nodes with >70 % posterior support for these branches. Nodes to the right of branches with <70 % support are left unlabelled. The bar graph indicates location probabilities of the node at the root of the tree (i.e., the most recent ancestor of all the sequences represented in the tree). Grey bars represent the probabilities obtained with randomization of the tip locations. Probable introduction events from Africa to Madagascar are indicated with blue arrows. (TIFF 1199 kb) Additional file 6: Figure S5. Maximum clade credibility tree constructed from the EACMV-like DNA-B dataset. Branches are coloured according to the most probable location state of the node on their right (i.e., the likely geographical location of the ancestral sequence represented by this node). The large black circle around one of the nodes indicates that the state probability at this node is less than 0.5 (i.e., there is less than 50 % confidence in the indicated location being the actual place where this ancestral sequence existed). The time-scale of evolutionary changes represented in the tree is indicated by the scale bar above it. Whereas filled circles that are associated with nodes indicate >95 % posterior probability support for the branches to their left, open circles indicate nodes with >70 % posterior support for these branches. Nodes to the right of branches with <70 % support are left unlabelled. The bar graph indicates location probabilities of the node at the root of the tree (i.e., the most common ancestor of all the sequences represented in the tree). Grey bars represent the probabilities obtained with randomization of the tip locations. Probable introduction events from Africa to the SWIO islands and Madagascar are indicated with respectively red and blue arrows. (TIFF 1441 kb) Additional file 7: Table S2. List of the sequences used in the analyses. (XLSX 67 kb)

## Abbreviations

ACMBFV: African cassava mosaic Burkina Faso virus
ACMV: African cassava mosaic virus
CMD: Cassava mosaic disease
CMG: Cassava mosaic geminiviruses
CMMGV: Cassava mosaic Madagascar virus
CP: Capsid protein
CR: Common region
DAPC: Discriminant analysis of principal components
EACMCV: East African cassava mosaic Cameroon virus
EACMKV: East African cassava mosaic Kenya virus
EACMMV: East African cassava mosaic Malawi virus
EACMV: East African cassava mosaic virus
EACMZV: East African cassava mosaic Zanzibar virus
SACMV: South African cassava mosaic virus
sPCA: Spatial principal components analysis
SWIO: South-West Indian ocean
