# Supplementary figures and images for: Divergent evolutionary and epidemiological dynamics of cassava mosaic geminiviruses in Madagascar
Source: BMC Evol Biol. 2016 Sep 6;16(1):182. doi: 10.1186/s12862-016-0749-2 (PMC5012068; doi:10.1186/s12862-016-0749-2)

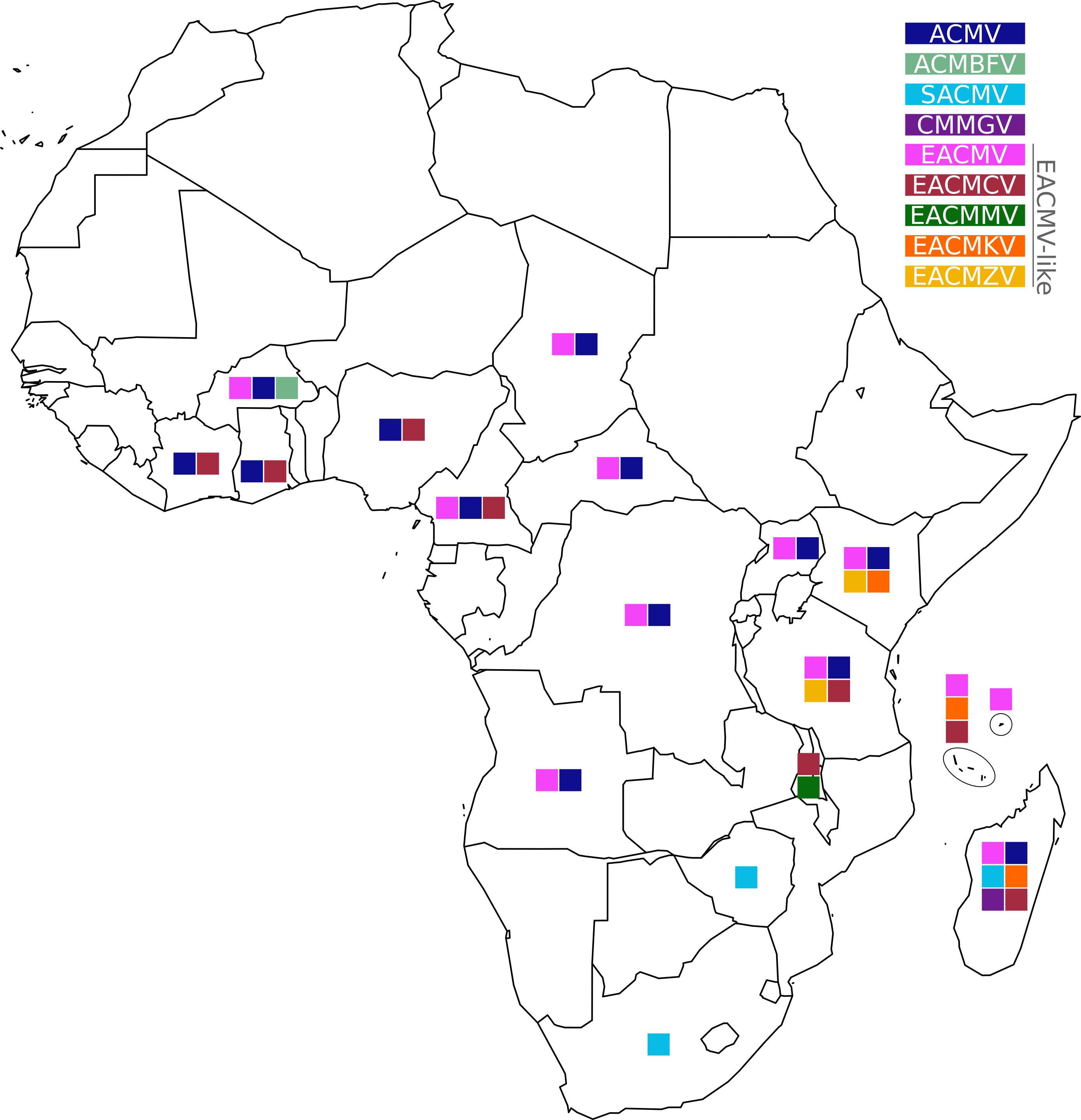

Supplement: Additional file 1: Figure S1. — Geographical map of African CMGs. The nine CMG species identified in Africa and on the SWIO islands are represented by a coloured circle in the countries where they have been isolated. (TIFF 600 kb) [file 12862_2016_749_MOESM1_ESM.tiff]

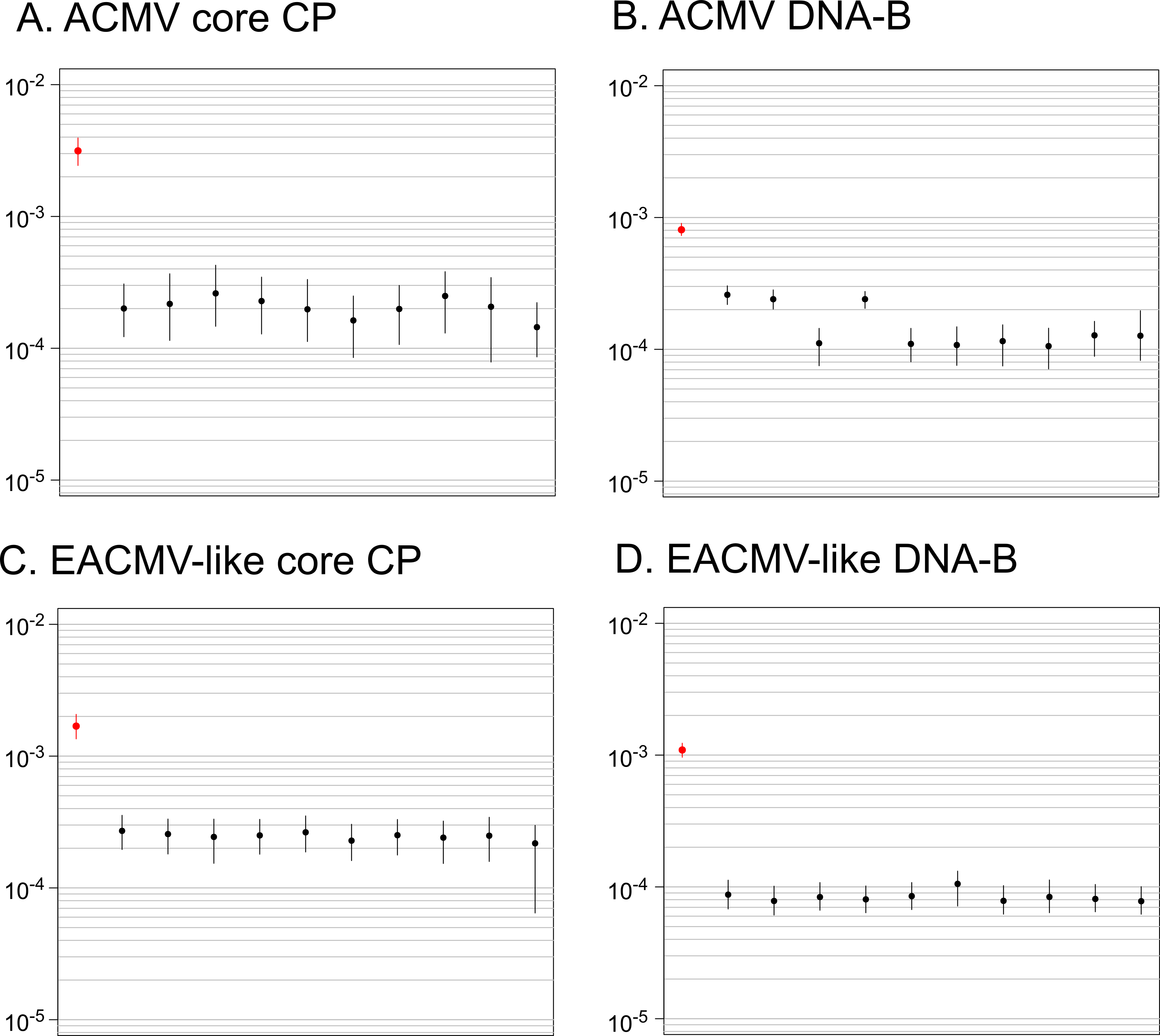

Supplement: Additional file 4: Figure S3. — Comparison of substitution rates inferred with tip-dates randomization. The substitution rates inferred by BEAST with and without randomization of the sequences sampling dates are indicated on a base-ten log scale for each dataset. Panel A: ACMV DNA-A core CP; Panel B: ACMV DNA-B; Panel C: EACMV-like DNA-A core CP; Panel D: EACMV-like DNA-B. Vertical lines represent the 95 % highest posterior density (HPD). Real datasets values and randomized datasets values are coloured respectively in red and black. (TIFF 349 kb) [file 12862_2016_749_MOESM4_ESM.tiff]

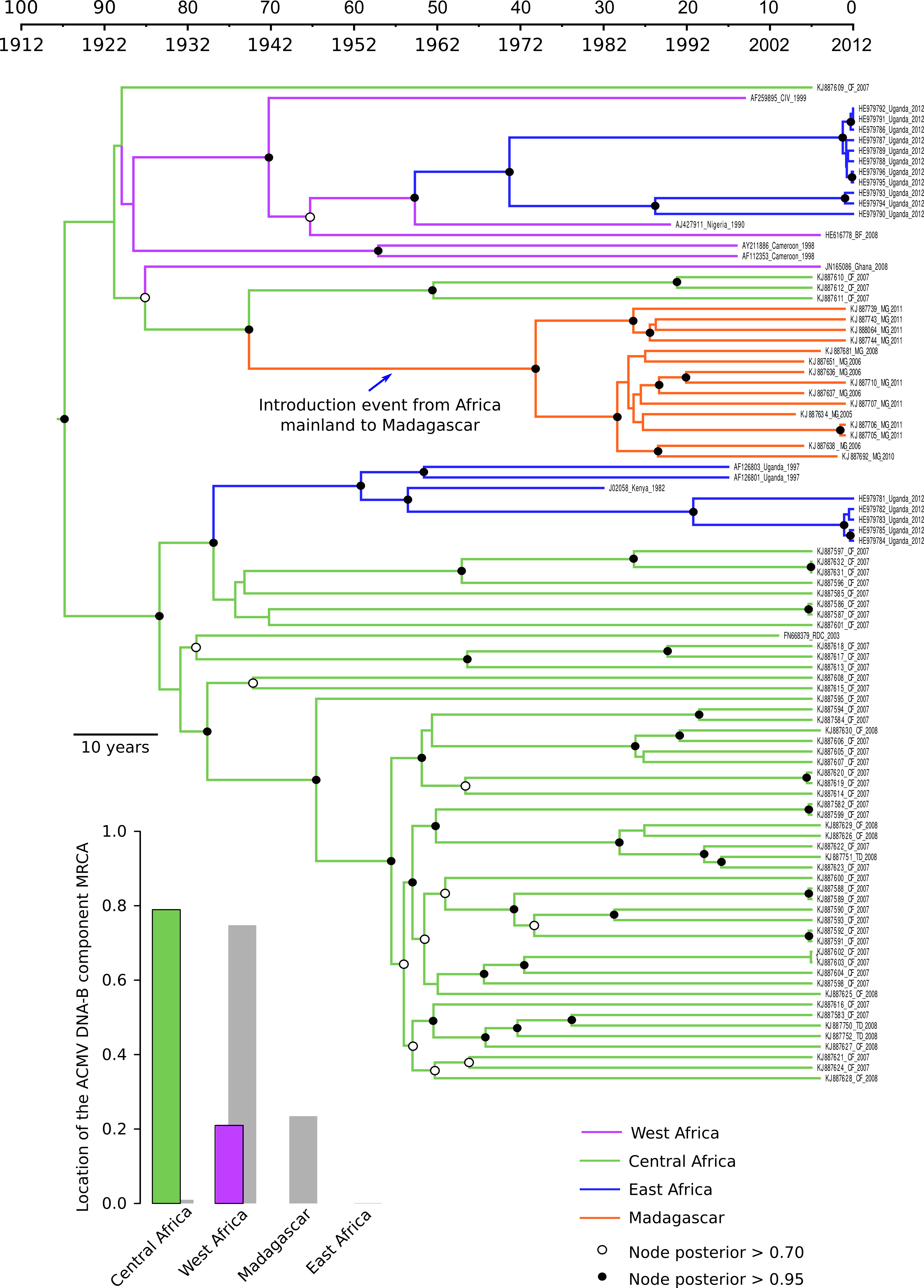

Supplement: Additional file 5: Figure S4. — Maximum clade credibility tree constructed from the ACMV DNA-B dataset. Branches are coloured according to the most probable location state of the node on their right (i.e., the likely geographical location of the ancestral sequence represented by this node). The time-scale of evolutionary changes represented in the tree is indicated by the scale bar above it. Whereas filled circles that are associated with nodes indicate >95 % posterior probability support for the branches to their left, open circles indicate nodes with >70 % posterior support for these branches. Nodes to the right of branches with <70 % support are left unlabelled. The bar graph indicates location probabilities of the node at the root of the tree (i.e., the most recent ancestor of all the sequences represented in the tree). Grey bars represent the probabilities obtained with randomization of the tip locations. Probable introduction events from Africa to Madagascar are indicated with blue arrows. (TIFF 1199 kb) [file 12862_2016_749_MOESM5_ESM.tiff]

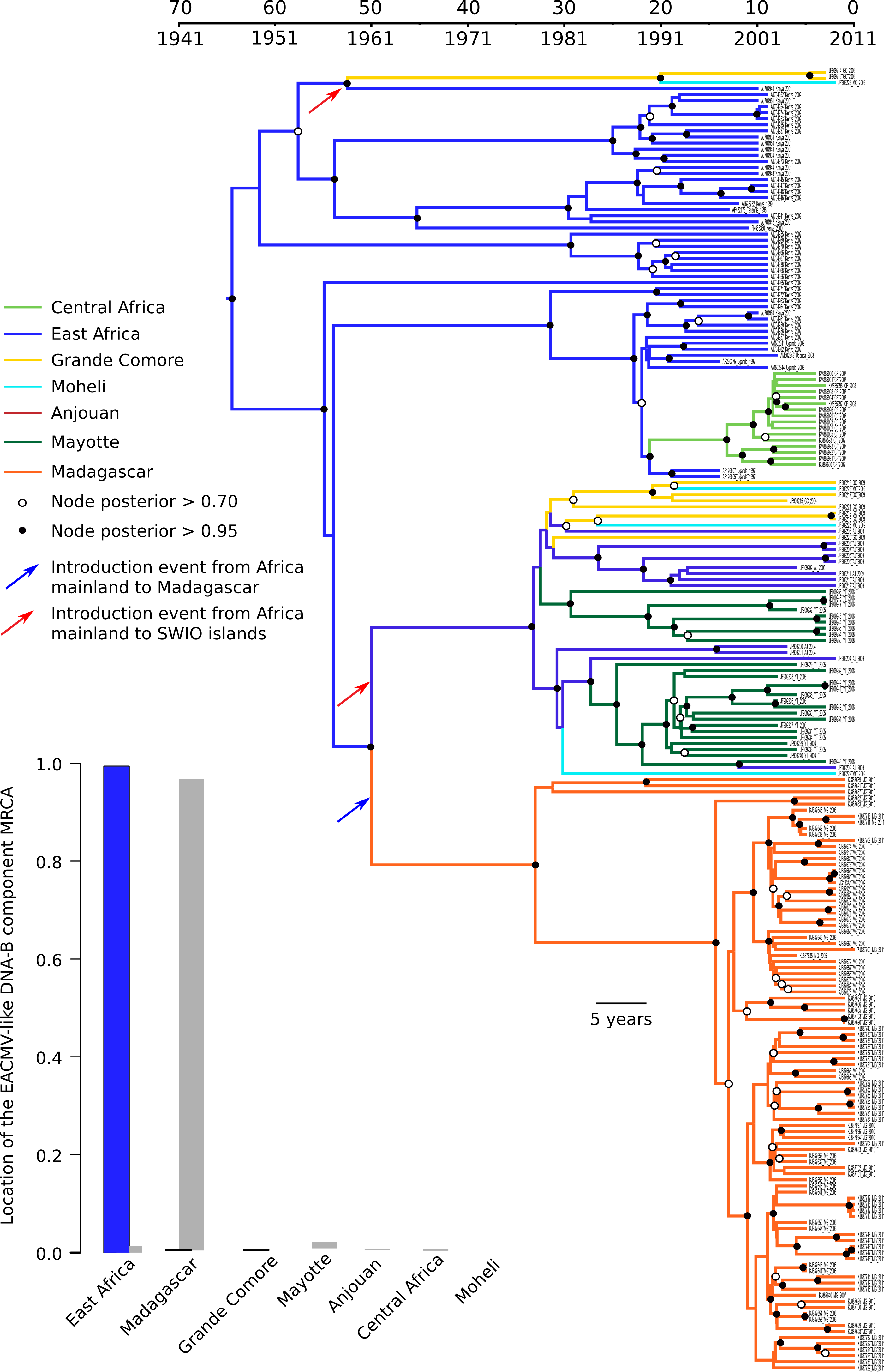

Supplement: Additional file 6: Figure S5. — Maximum clade credibility tree constructed from the EACMV-like DNA-B dataset. Branches are coloured according to the most probable location state of the node on their right (i.e., the likely geographical location of the ancestral sequence represented by this node). The large black circle around one of the nodes indicates that the state probability at this node is less than 0.5 (i.e., there is less than 50 % confidence in the indicated location being the actual place where this ancestral sequence existed). The time-scale of evolutionary changes represented in the tree is indicated by the scale bar above it. Whereas filled circles that are associated with nodes indicate >95 % posterior probability support for the branches to their left, open circles indicate nodes with >70 % posterior support for these branches. Nodes to the right of branches with <70 % support are left unlabelled. The bar graph indicates location probabilities of the node at the root of the tree (i.e., the most common ancestor of all the sequences represented in the tree). Grey bars represent the probabilities obtained with randomization of the tip locations. Probable introduction events from Africa to the SWIO islands and Madagascar are indicated with respectively red and blue arrows. (TIFF 1441 kb) [file 12862_2016_749_MOESM6_ESM.tiff]
